# Nurse-Led Medicines' Monitoring for Patients with Dementia in Care Homes: A Pragmatic Cohort Stepped Wedge Cluster Randomised Trial

**DOI:** 10.1371/journal.pone.0140203

**Published:** 2015-10-13

**Authors:** Susan Jordan, Marie Ellenor Gabe-Walters, Alan Watkins, Ioan Humphreys, Louise Newson, Sherrill Snelgrove, Michael S Dennis

**Affiliations:** 1 College of Human and Health Sciences, Swansea University, Swansea, Wales; 2 College of Medicine, Swansea University, Swansea, Wales; University Hospital Basel, SWITZERLAND

## Abstract

**Background:**

People with dementia are susceptible to adverse drug reactions (ADRs). However, they are not always closely monitored for potential problems relating to their medicines: structured nurse-led ADR Profiles have the potential to address this care gap. We aimed to assess the number and nature of clinical problems identified and addressed and changes in prescribing following introduction of nurse-led medicines’ monitoring.

**Design:**

Pragmatic cohort stepped-wedge cluster Randomised Controlled Trial (RCT) of structured nurse-led medicines’ monitoring *versus* usual care.

**Setting:**

Five UK private sector care homes

**Participants:**

41 service users, taking at least one antipsychotic, antidepressant or anti-epileptic medicine.

**Intervention:**

Nurses completed the West Wales ADR (WWADR) Profile for Mental Health Medicines with each participant according to trial step.

**Outcomes:**

Problems addressed and changes in medicines prescribed.

**Data Collection and Analysis:**

Information was collected from participants’ notes before randomisation and after each of five monthly trial steps. The impact of the Profile on problems found, actions taken and reduction in mental health medicines was explored in multivariate analyses, accounting for data collection step and site.

**Results:**

Five of 10 sites and 43 of 49 service users approached participated. Profile administration increased the number of problems addressed from a mean of 6.02 [SD 2.92] to 9.86 [4.48], effect size 3.84, 95% CI 2.57–4.11, P <0.001. For example, pain was more likely to be treated (adjusted Odds Ratio [aOR] 3.84, 1.78–8.30), and more patients attended dentists and opticians (aOR 52.76 [11.80–235.90] and 5.12 [1.45–18.03] respectively). Profile use was associated with reduction in mental health medicines (aOR 4.45, 1.15–17.22).

**Conclusion:**

The WWADR Profile for Mental Health Medicines can improve the quality and safety of care, and warrants further investigation as a strategy to mitigate the known adverse effects of prescribed medicines.

**Trial Registration:**

ISRCTN 48133332

## Introduction

The adverse effects of prescribed medicines are a public health problem, in the UK[1–4], and worldwide[5,6]. Adverse drug reactions (ADRs)7 are responsible for: 20.8% (60/290) of preventable emergency re-admissions within one year of discharge[8]; 4–6% of UK hospital bed occupancy[9]; 10% (68/678) hospitalisations in US Veterans’ Affairs Medical Centres[10]; 3.7% hospital admissions globally[11]; an increasing number of UK hospital admissions[12]; £1–2.5bn NHS costs annually[13] and ~$30bn expenditure each year in the USA[14]. Although preventable ADRs account for 5–8% of all hospital admissions[15], adverse consequences of untreated conditions are equally common in primary care[16]. Enhanced patient monitoring might prevent many of these problems[6,17–21], and our nurse-led intervention has improved care in several clinical settings[22–25].

We focus on three key medicine groups: antipsychotics, anti-epileptics, and antidepressants, which are often prescribed to people living with dementia, either for the management of associated behavioural and psychological symptoms or the frequently accompanying co-morbid conditions of depression and epilepsy. However there are concerns relating to the use of these medicines because of their side-effect profiles. Whether to manage symptoms of dementia or co-morbidities, 25–50% of people with dementia receive antipsychotics[26–28]; one third of care home residents receive antidepressants[29]; and 10–20% of people with Alzheimer’s disease require anti-epileptics[30]. Consequently, many older people, particularly those with dementia, are vulnerable to the adverse effects of mental health medicines[31,24], as evidenced by current prescribing trends[32]. The Protean ADRs attributed to mental health medicines include worsening cognitive impairment, aggression/ restlessness, sedation, falls, bleeding, and changes in cardiovascular and gastro-intestinal function, as detailed on the WWADR Profile[24] (S1 Appendix). There is particular concern over the use of antipsychotic medication in people living with dementia, with only modest evidence to suggest clinical improvement but increasing risk of adverse health outcomes and mortality[25].

Despite widespread acknowledgment of the ‘ADR problem’[33,34], no recommendations on implementation of medicines’ monitoring procedures are offered. While the UK’s National Institute for Health and Care Excellence (NICE) [35] specify parameters for monitoring, neither they nor leading reviews[19] nor reports[3,4] offer monitoring strategies. Written information, while not harmful, is not well regarded by service users, and on its own is insufficient[36,37]. However, some primary care physicians undertake medication review only if offered suitable financial incentives, including upfront payment of £350[38], and evidence from pharmacist-led interventions is equivocal[39] and described as ‘low quality’[40,16] or uncertain[19,41]. Accordingly, our West Wales ADR (WWADR) Profiles are designed to use an alternative link in the ‘medication chain’, and to be completed by nurses, who are usually the professionals with the most intimate knowledge of patients. The Profiles aim to minimize ADRs without compromising the beneficial effects of medicines[22–25,42]–congruent with recent ‘calls to action’[2–4,43]—and promote guideline adherence[15,35]. However, the WWADR Profile for Mental Health medicines has not been tested in clinical trials

We aimed to assess 1) clinical impact 2) potential cost impact and 3) any harms of nurse-led medicines’ monitoring using the WWADR Profile for Mental Health Medicines[24,42]. Our objectives were to test whether, at individual participant level, introduction of the Profile affected: the number and nature of problems identified and addressed; prescribing; dementia severity; potential service costs and harms.

## Methods

### Design

In this cohort stepped wedge cluster randomised trial (SW-CRT), five sites (care homes) were randomised to five sequential steps or time points. There were equal numbers of observations with and without the Profile over the six months of the trial. The cohort SW-CRT design strengthens the traditional cluster randomised controlled trial (RCT): all participants receive the intervention, reducing ‘resentful demoralisation’; at each step, sites who receive the intervention act as cross-sectional controls for those who had not; each participant acts as their own longitudinal control, allowing ‘before and after’ comparison[44,45]; and institution level adoption of the Profile simulates a policy-level intervention. Because all participants cross-over to the intervention by the end of the trial, cohort SW-CRTs are recommended for testing patient safety interventions[46], likely to be beneficial[47], and unlikely to cause harm. (The CONSORT checklist is appended [S1 CONSORT Checklist]) [48]. The authors confirm that all ongoing and related trials for this intervention are registered (ISRCTN 48133332). Prior to participant recruitment in May, we recruited and trained the nurses involved in the project.

The SW Wales NHS Research Ethics committee (REC) approved the study (reference: 13/WA/0067, 10th April 2013). The protocol is available (S1 Protocol).

#### Participants

Recruitment and follow-up of participants was undertaken May to October 2013 in five sites with 181 service users. The sites were dispersed across South West Wales and run by different private sector organisations. In all sites, prescription and administration of all medicines were documented on paper-based medicines’ administration record (MAR) charts, which were the responsibility of qualified nurses. Prescriptions were issued by primary care practitioners and consultant-led secondary care services. Medicines were dispensed by community pharmacists in original packaging. Each site had its own record keeping format. Patient records, including medication administration records, were regularly inspected to ensure they met the standards of the Care and Social Services Inspectorate Wales (CSSIW). In care homes, medication records include preparations for minor ailments, which are normally purchased by ambulatory service users, and not necessarily recorded.

Individual participants’ inclusion criteria were: resident at the site; currently taking antipsychotics and/or anti-epileptics and/or antidepressants; willing and able to give informed, signed consent themselves, or where capacity was lacking, a guardian or consultee was willing to give informed, verbal assent to review of documentation. We excluded those: aged <18 years; not well enough to participate, according to the clinical judgement of their nurses.

#### Intervention

The West Wales ADR Profile for Mental Health Medicines[24] was introduced into the five sites at staggered monthly intervals or steps, for completion with all eligible participants. Once the Profile and guidelines had been introduced, and nurses trained (by SJ, MG), nurses were asked to re-administer it every step as part of usual care. From previous work[24], we anticipated that Profile completion would take 15–25 minutes.

The Profile aims to: alleviate under-reporting of adverse effects of prescribed medicines, facilitate shared decision-making with service users and within the multi-disciplinary team[15,49], and identify problems that merit attention irrespective of aetiology. The Profile contains a structured template of 82 items to be completed over several interviews, if needed[24], with comprehensive guidelines[42], to ensure unified recording of all information pertinent to potential adverse effects of medicines commonly prescribed for mental health conditions[24,42] (S1 and S2 Appendix). The profile was developed for administration during routine nursing care[24,42,50–52], incorporating ADRs documented in formularies[34,53], and manufacturers’ literature. Inter-rater reliability for the items’ kappa values ranged 0.44–1.00, with observational items generally having lower values[42].

#### Outcomes

The primary outcome was the number and nature of problems found and addressed, described under ‘clinical impact’ in ‘data collection’, below. Secondary outcomes were changes in individual problems, prescribing, disease severity, and potential costs and harms of the intervention.

#### Sample size

In our feasibility before and after study[24] a mean of 3.0 [SD 5.4] more problems were addressed following administration of the Profile, and the intra-cluster correlation coefficient (ICC) was close to zero. To investigate whether this improvement would be replicated in a larger sample would require a total sample of 28, with 80% power, 5% alpha, two sided[54]. With 10 participants in each cluster, an ICC of 0.05, and a design effect of 1.45, we calculated that 41 participants were needed[55]. We planned to recruit 50 participants from 5 sites, and anticipated 10% loss to follow up over 6 months.

#### Recruitment

Ten sites (care homes) were approached by the research team. Sites were identified from the list of those working with the University, and telephoned for an appointment to explain the study and request participation pre-randomisation. All service users in participating sites were assessed for eligibility and those meeting the inclusion criteria were approached by their nurses.

#### Randomisation

The five sites were randomised by the Swansea Clinical Trials Unit (SCTU) to determine at which of the five steps the Profile would be introduced in each site. Sites were informed one month before introduction of the intervention.

#### Data Collection

Participants’ records were reviewed pre-intervention and at each of 5 subsequent data collection steps. One month between each step minimised the impact of delayed treatment effects on study power[56]. We (MEG, SJ, LN, SS) extracted data from participants’ case notes to identify compliance with the intervention and obtain information on trial outcomes and objectives:

1. Clinical impact was measured by:
  - Total number of problems documented as present and addressed at each data collection step (primary outcome), as interval variables. The presence or absence of each possible ADR and whether it was addressed were noted as binary variables (S1 Appendix).
  - The nature of specific benefits of the intervention, including: actions taken to reduce ADRs listed on the Profile (S1 Appendix), such as monitoring vital signs, intake and medicines’ administration[3,4], and pain management, based on previous work[24] Pain is particularly important, as effective pain management improves the treatment of behavioural difficulties and agitation in people with dementia in care homes[57]. Dementia, sedation, antipsychotics and antidepressants are also important risk factors for falls and injuries[30,34,58,59,60,61,62]. Case reports are included to capture the burden, severity or nature of the problems. The presence or absence of each problem and actions taken to address the problem were captured as binary variables, and the proportion of participant records where these were documented was calculated with participants and steps as the denominator (total 249). Case reports are included to capture the burden, severity and nature of the problems.
  - Changes in prescription regimens as documented in administration records and referral letters. Numbers, names and doses of medicines prescribed were recorded, and binary variables ‘any change in regimen’ and ‘reduction of medicines associated with mental health’ (antipsychotics, antidepressants, anitepileptics, benzodiazepines and antimuscarinics prescribed to manage the adverse effects of antipsychotics) were calculated at each step.
  - Measures of dementia and illness severity: the Bristol Activities of Daily Living (ADL)[63] scale for functional ability and MOUSEPAD[64] for psychopathology, as total scores recorded by nurses at every data collection step, and treated as interval variables. Increasing scores indicated increased dependency / dementia severity.
2. Estimated potential economic costs of delivering the intervention
3. Evidence that the Profile had harmed or burdened participants as captured in participants’ records, including adverse event forms, and reports from care providers.

#### Analysis

Data were entered into SPSS (version 20, IBM Corp. in Armonk, NY) for main analyses and into R (R Foundation for Statistical Computing, Vienna, Austria) for confirmatory analyses. Problems addressed were listed. Analysis was based on participant level data, clustered within sites. Outcome variables were analysed using generalized linear mixed effects models with an indicator of profile use, baseline data on age, number of medicines prescribed, prescription of antipsychotics, antiepileptics, antidepressants and SSRIs, (detailed in Table 1), and data collection step considered as fixed explanatory variables. We considered separate models for each variable of interest. We used normal models for interval variables and binary logistic regression models for binary data. In each model, site was entered as a random effect variable; this formally accommodated the observed ICCs in our data. The effect of the intervention is reported as adjusted mean differences (β for interval variables) or adjusted odds ratios (aOR for binary variables) taken from models which retain statistically significant covariates and factors from the list above. Inferential statistics were confined to composite outcomes (numbers of problem found and addressed, changes in prescribing, measures of illness severity) and examples of the nature of the problems addressed selected for clinical importance (pain, falls, sedation).

**Table 1:** Recruitment, Retention, Demographics and Prescription medicines at study entry.

	Site 1	Site 2	Site 3	Site 4	Site 5	Full sample
**Recruitment**						
Number eligible	10	8	9	12	10	49
Number starting trial	10	8	5	10	10	43
**Retention**						
Number completing	10	8	5	9	9	41
**Demographics**						
Number female	3	3	5	7	7	25
Age: mean [SD] in years	72.60 [7.89]	69 [12.14]	87.40 [6.99]	85.90 [6.49]	81.00 [9.65]	78.7 [11.00]
Age: median [25^th^–75^th^ centile]	71.5 [65.5–80.75]	63 [60.25–83.25]	88 [82–92.5]	87 [81–91.25]	82.5 [71.5–88.5]	80 [67–88]
Range (min-max)	63–84	58–88	76–95	75–96	66–94	58–96
Number registered for nursing care*	5	6	0	0	5	16
**Prescribed medicines**						
Mean [SD] number of medicines**/ participant	6.8 [3.4]	11.1 [3.9]	9.6 [1.1]	9.1 [4.2]	10.6 [3.1]	9.3[3.7]
Median [25^th^–75^th^ centile] number of medicines/participant	6 [4–9.3]	10.5 [9–13.3]	10 [8.5–10.5]	8 [6.5–11.8]	10.5 [7.5–13.5]	9 [7–11]
Range (min-max)	2–13	6–19	8–11	4–18	7–15	2–19
Number of participants prescribed:						
Antipsychotics	5	5	3	6	2	21
Antidepressants (not SSRIs)	0	2	4	5	2	13
Antiepileptics	1	7	2	6	1	17
SSRIs	3	2	0	2	7	14

* Categorisation follows a needs assessment. Service users are assessed by NHS nurses and designated as needing nursing care or residential care. Assessments are usually based on the Royal College of Nursing’s (2004) ‘Nursing assessment and Older People An RCN Toolkit’. London, RCN: http://www.rcn.org.uk/__data/assets/pdf_file/0010/78616/002310.pdf

** Any combination preparations were counted as a single item. Enumerating the active ingredients of each product would have been impractical, particularly for antacids and multivitamins.

Some problems could not be addressed within the month between data collection steps, most particularly visits to opticians and dentists, where NHS access can take several weeks. To account for the delayed effects of treatment, we analysed data on opticians’ and dentists’ visits from first and last collection steps, thus effectively undertaking ‘before and after’ analyses of numbers accessing these services within the last year; we retained all other aspects of the modelling strategy, including a random effects variable for site. We undertook standard model and residual diagnostics where appropriate; presentation of the analyses reflects joint decisions by two authors. Case vignettes are presented for illustration.

### Ethics

The study was explained to all potential participants and, where possible, their families, supplemented by written information, in English and Welsh. Residents’ or consultees’ consent for researchers to review participants’ notes for information directly relevant to medicines’ use was sought by qualified nurses, who were familiar with the Mental Capacity Act (2005)[65] and employed by the site. Since this was a cluster RCT[66,67], and the research only involved excerpting non-identifiable data from records, where the participant lacked capacity, the REC considered it appropriate for qualified site staff to take verbal consent/ assent, witnessed by a signatory outside the research team[7:16,68:34]. Participants’ General Practitioners (GPs) were informed of the project by letter. Usual standard care was delivered throughout. To cover the time spent in recruiting participants and administering the measures of functioning and dementia severity, sites received honoraria. Honoraria were not intended to cover administration of the Profile, as the questions posed were those that should be asked in routine care.

## Findings

**Fig 1:**
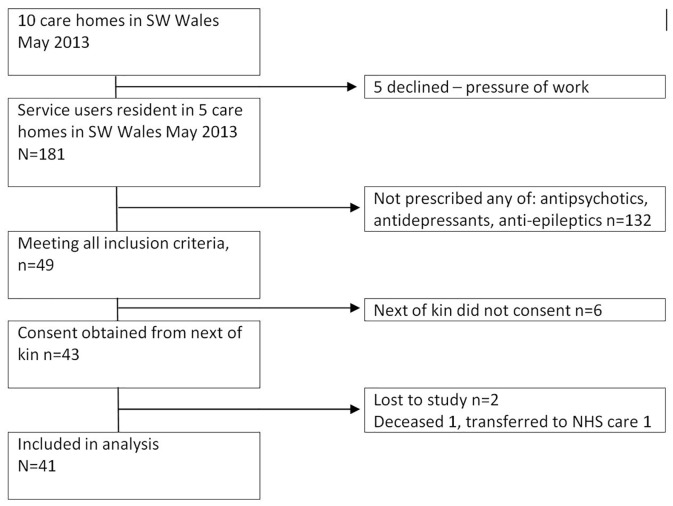
Numbers in the analysis.

Five of 10 sites declined to participate; ‘pressure of work’ was the only explanation offered. Of the 181 service users, 49 met inclusion criteria, and 43 consented to participate. In care home 5, 2 joined late. Nurses indicated that all participants lacked capacity to consent, and consent was obtained from consultees. We retained 41 of the 43 service users: one died, and one did not return from hospital, all before introduction of the Profile. Recruitment is summarized in Fig 1 and Table 1, and retention in Table 2.

**Table 2:** Patient monitoring: number of participants with documentation of each problem. 2 participants joined late and 2 were lost to the study.

		Step 1 baseline no Profiles n = 41			Step 6 all participants use Profile n = 41		
	Acceptable ranges*	Problem n(%)	No problem n(%)	Not monitored n(%)	Problem n(%)	No problem n(%)	Not monitored n(%)
**Vital signs**							
Heart rate	55–90bpm	0	28 (68.3)	13 (31.7)	2 (4.9)	30 (73.2)	9 (22.0)
Heart rhythm	No irregularity	0	1 (2.4)	40 (97.6)	1 (2.4)	21 (51.2)	19 (46.3)
BP– 2 position for postural hypotension	Systolic drops <20mmHg or 10% on standing	8 (19.5)	23 (56.1)	10 (24.4)	7 (17.1)	25 (61.0)	9 (22.0)
Weight	Change of <2.4kg in 1 month	19 (46.3)	20 (48.8)	2 (4.9)	17 (41.5)	22 (53.7)	2 (4.9)
Girth	<88cms in women, <102cms. in men	0	0	41 (100)	14 (34.1)	4 (9.6)	23 (56.1)
Temperature	36.8 ± 0.4°C	4 (9.6)	6 (14.6)	31 (75.6)	3 (7.3)	18 (43.9)	20 (48.8)
Oxygen saturation	≥97%	0	0	41	10 (24.4)	18 (43.9)	13 (31.7)
**Intake**							
Fluids	>1.2L + 1L taken within solid food	1 (2.4)	14 (34.1)	26 (63.4)	7 (17.1)	33 (80.5)	1 (2.4)
Missed meals	≤1 meal unfinished / day	6 (14.6)	17 (41.5)	18 (43.9)	14 (34.2)	25 (61)	2 (4.9)
**Medicines’ administration**							
Missed doses	<2 doses of prescribed medicines missed over any period of 7 days in the last month (including refusal)	1 (2.4)	5 (12.2)	35 (85.4)	6 (14.6)	35 (85.4)	0
Regular administration	Regular medication taken at same time each day	0	6 (14.6)	35 (85.4)	1 (2.4)	39 (95.1)	1 (2.4)

* Guidelines appended to the Profile offered definitions of acceptable ranges of measurement and standards. Guidelines are available on request.

At trial entry, 27/43 (62.8%) participants were receiving residential care, and 16 nursing care; 25/43 (58.1%) were female. Participants’ ages ranged 58–96, mean 78.7 [SD 11.00], median 80 [IQR 67–88].

Of the 43 participants, 39 had a recorded primary diagnosis of dementia (including alcohol-related, vascular, Alzheimer’s, fronto-temporal), two were diagnosed with Korsakoff’s syndrome, and two with depression, but not dementia. A diagnosis of depression was recorded for 7 participants (five with co-morbid depression where primary diagnosis was dementia), and 27 were prescribed antidepressants. Prescribing patterns were not uniform in the 5 sites: more participants were prescribed antiepileptics in site 2; site 5 used more SSRIs and fewer antipsychotics (Table 1). Participants were prescribed between 2 and 19 medicines, (median 9 [7–11], mean 9.3 [SD 3.7]), including treatments for physical health conditions.

Profile completion and all cross-over to Profile use was as scheduled. Laboratory test results were often unavailable, as they were held by participants’ GPs. Monitoring of vital signs, food and fluid intake and medicines’ administration increased on introduction of the Profile, and was sustained at the close of the trial. Before Profile introduction, fluid and food intake and medicines’ administration were monitored for ~50% participants; this rose to >95% of participants when all sites used the Profile. However, compliance with vital signs’ monitoring was incomplete, particularly for heart rhythm, girth and temperature (Table 2). Some nurses reported insufficient time to complete physiological measurements. Nine participants were unable to stand, precluding measurement of standing BP.

### 1. Clinical impact

#### a. Problems found and addressed

More problems were found and actions taken when the Profile was used (Tables 3 and 4). More problems were found than were addressed. Most problems were actioned more frequently with than without the Profile, particularly balance/ co-ordination, cognitive decline, violence, aggression, mood fluctuations, behaviour problems, restlessness, confusion, apathy, hallucinations, sedation, headache, hearing problems, hypersalivation, emesis, missed meals, inadequate fluid intake, vision problems, pain and missed medicines; the exceptions were changes in weight, insomnia and bowel control. Typically, nurses modified care plans and made referrals (Table 5). In some cases, problems such as incontinence, were long-standing and considered intractable. The sharp and sustained increase in problem detection at first use of the Profile in all sites (Table 3) was not reflected in actions taken to address problems in all sites: in sites 2,4 and 5 the mean number of problems addressed more than doubled, whereas little change was seen in site 1, and there was a slight decrease in site 3 (Table 4).

**Table 3:** Total number of problems found per participant at each step and in each site. Bold text indicates roll-out of medicines’ monitoring and Profile administration 1 month before these data collection points (occasions when researchers extracted data from participants’ records). n = number of service users in the site. One participant from site 4 passed away between steps 3 & 4. One participant from site 5 was hospitalised between steps 3 & 4. Participants joined site 5 at steps 2 and 3. Problems explored are listed in Table 5 and on the Profile, S1 appendix. Fuller versions of these tables, including medians and 25^th^ = 75^th^ centiles are in S1 and S2 Tables.

	Total number of problems found					
Site	Step 1.	Step 2.	Step 3.	Step 4.	Step 5.	Step 6.
**5:** n	8	9	10	9	9	**9**
Mean [SD]	6.38 [2.00]	6.78 [1.92]	7.50 [2.32]	7.70 [3.65]	7.40 [3.89]	**16.50 [6.59]**
Full range	5–11	4–11	5–12	1–14	1–16	**1–24**
**4:** n	10	10	10	9	**9**	**9**
Mean [SD]	9.10 [3.81]	6.20 [2.49]	7.00 [2.63]	4.56 [2.30]	**18.33 [7.57]**	**19.11[6.79]**
Full range	2–14	2–10	2–11	2–8	**7–27**	**0–5**
**3:** n	5	5	5	**5**	**5**	**5**
Mean [SD]	8.20 [4.09]	6.60 [2.30]	6.80 [4.32]	**10.60 [2.07]**	**9.40 [1.14]**	**12.20 [2.39]**
Full range	5–15	3–9	1–13	**8–13**	**8–11**	**9–15**
**2:** n	8	8	**8**	**8**	**8**	**8**
Mean [SD]	8.13 [3.72]	5.38 [3.58]	**15.75 [6.94]**	**12.75 [5.90]**	**12.88 [6.31]**	**14.38 [5.73]**
Full range	3–13	1–10	**5–28**	**5–24**	**5–22**	**9–28**
**1:** n	10	**10**	**10**	**10**	**10**	**10**
Mean [SD]	10.30 [2.21]	**17.30 [6.08]**	**17.30 [6.09]**	**17.00 [5.25]**	**16.50 [5.19]**	**17.10 [4.61]**
Full range	7–14	**10–25**	**6–25**	**6–26**	**5–23**	**6–23**

**Table 4:** Total number of problems addressed per participant at each step in each site. Bold text indicates roll-out of medicines’ monitoring and Profile administration 1 month before these data collection points (occasions when researchers extracted data from participants’ records). n = number of service users in the site. One participant from site 4 passed away between steps 3 & 4. One participant from site 5 was hospitalised between steps 3 & 4. Participants joined site 5 at steps 2 and 3. Problems explored are listed in Table 5 and on the Profile, S1 appendix. Fuller versions of these tables, including medians and 25^th^ = 75^th^ centiles are in S1 and S2 tables.

	Total number of problems addressed					
Site	Step 1.	Step 2.	Step 3.	Step 4.	Step 5.	Step 6.
**5:** n	8	9	10	9	9	**9**
Mean [SD]	4.25 [1.67]	4.89 [1.90]	5.30 [2.41]	5.00 [2.83]	4.40 [2.22]	**10.70[4.97]**
Full range	3–8	3–9	3–10	1–12	1–8	**91–21**
**4:** n	10	10	10	9	**9**	**9**
Mean [SD]	8.50 [3.44]	5.90 [2.47]	6.60 [2.27]	4.44 [2.24]	**12.00 [4.61]**	**12.78 [4.68]**
Full range	2–13	2–10	2–9	2–8	**6–19**	**6–19**
**3:** n	5	5	5	**5**	**5**	**5**
Mean [SD]	7.00 [2.92]	5.80 [1.92]	6.00 [4.18]	**4.60 [1.34]**	**4.60 [1.52]**	**6.20 [2.17]**
Full range	4–11	3–8	1–12	**3–6**	**3–7**	**0–6**
**2:** n	8	8	**8**	**8**	**8**	**8**
Mean [SD]	7.25 [3.37]	5.00 [3.12]	**11.50 [4.38]**	**8.88 [4.61]**	**7.88 [5.17]**	**10.13 [5.19]**
Full range	2–11	1–8	**3–18**	**2–17**	**3–16**	**4–22**
**1:** n	10	**10**	**10**	**10**	**10**	**10**
Mean [SD]	9.30 [2.63]	**8.60 [2.99]**	**9.80 [3.62]**	**10.10 [3.41]**	**10.80 [4.57]**	**11.70[4.72]**
Full range	5–13	**5–14**	**3–14**	**4–16**	**3–18**	**4–20**

**Table 5:** Problems addressed with and without the Profile: total numbers and examples. Visits to dentists and opticians were compared ‘before and after’ (see analysis and Table 6). ADLs—activities of daily living.

Problem	*Using Profile -124 data collection points*	*Without the Profile—125 data collection points*	*Examples of Typical actions*
Heart rate	1	1	GP informed
Irregular rhythm	*0*	*0*	None when identified
BP	6	5	Daily recordings. GP informed.
Weight / BMI	19	39	Care plan to encourage/ discourage intake.
Girth	11	0	Care plan for weight loss
Temperature	3	3	Additional blankets offered
oxygen sats	8	0	GP contacted
ECG	0	0	1 recording, no problems
Hand tremor	2	0	Care plans for Parkinson’s and carpal tunnel
Tongue tremor	0	0	No actions
Feet shuffling	5	2	Care plans to address risk of falls
Abnormal movements	2	2	Care plans and medication
Posture abnormal	5	5	occupational therapy referral
Gait abnormal on walking	9	11	occupational therapy referral
Balance abnormal/ co-ordination poor & interferes with ADLs	44	22	Care plans for staff to assist with mobility and address risk of falling
Bleeding or bruising	12	7	Charting and monitoring. Care plan for polyps.
Feeling the cold	2	0	Extra blankets
Cognitive decline	84	44	Care plans for staff to offer assistance
Convulsions	14	4	Care plan updated. Specialist referral.
Self-harm	2	5	Care plan updated
Violence	17	10	Reports to mental health team
Aggression	49	40	Reported to mental health team, and medication to be continued.
Irritability	38	2	Care plan to improve explanations. Linked to constipation and UTI.
Mood fluctuations	45	23	Care plans to reassurance and encourage. Medication review and changes.
Agitation, anxiety, nervousness	67	52	Care plan to reduce anxiety with music and reassurance. Medical review.
Behaviour problems e.g. ‘sexualised behaviour’	35	11	Care plans to offer reassurance. Mental health review.
Restlessness or pacing	20	4	Mental health team to review patient and /or medicines.
Hyperactivity	3	2	Care plan modified.
Panic attacks	2	1	Care plan for management.
Confusion	106	57	Care plan for orientation, GP or mental health referral. MSU for analysis
apathy, low energy	21	2	Encourage intake. GP review.
Hallucinations, vivid dreams	21	8	Care plan to monitor and reassure. Mental health review. UTI treatment.
Sleep problems/ insomnia	17	24	Care plan to deal with wakefulness.
Sedation	16	3	GP referral. Omission of mid-day sedation
Diszziness	6	7	Risk of falls assessed. BP monitored.
Falls	32	34	Care plans for assistance, hoists, lighting at night, mobilising. GP referral.
Headache, migraine	14	0	Analgesia
Tinnitus/hearing problems	9	0	Encouraged to wear hearing aid. Syringing.
Tingling/pins & needles	0	1	Referred to doctor
Urination	87	84	Continence management. Care plan for retention management. Medical review.
UTI	28	29	Antibiotics
Reproductive system e.g. polyps, behaviour change	8	7	GP referral and directed management
Chest pain	2	0	GP referral
Short of breath	5	1	Managed as asthma or chest infection
Hypersalivation/	5	0	Monitored for chest infection. Hyoscine patch.
Nausea / vomiting	8	0	GP or dietician referral. PRN medicines.
Appetite/ taste changes	10	2	Dietician referral. Care plan for assistance with feeding.
Bowel control/ diarrhoea	47	59	Care plans for incontinence. Stool charts.
Constipation	13	12	Care plans to monitor. Laxatives.
Rash (+/- itching)	22	21	Creams applied. GP referral.
Swelling/ oedema / pressure areas	20	16	Waterlow scores, GP referral
Sweating / pressure areas	2	0	Enhanced skin care in care plan
Injection site e.g. pain	0	0	No injections
Missing any meals or leaving them unfinished more than once a day	44	19	Care plan to monitor or for soft diet or frequent small meals or offer alternatives e.g. milk. Dietician referral.
‘Snacking’ or eating between meals	13	0	Low fat diet.
Drinking 1 pint or more of milk or soya milk per day. This includes milk added to cereal & hot drinks	3	0	Milk drinks to be offered between meals. Soya offered.
Vitamin D intake adequate (time in sunlight, eats oily fish)	3	0	Diet updated.
Eating fruit or vegetables every day	4	0	Care plan to encourage
Drinking more than 2 litres, or 6–8 cups, per day. This includes water, tea, coffee or squash	11	4	Care plan to encourage and monitor
Are drinks sugar free?	4	0	Access to sugar-free drinks
Swallowing difficulties	9	8	SALT referral, assistance with eating.
Indigestion or heartburn	3	0	GP referral. Medication.
Problems with teeth or dentures	8	2	Mouth care. Dental referral.
Dry mouth	4	0	Mouth care
Halitosis	1	0	Dental referral
Vision problems or dry eyes	38	20	Optician referral, gel for dry eyes, large print
Is sunscreen available?	0	0	Not used
Is it applied evenly?	0	0	Not used
Dark glasses worn in bright sunlight?	4	0	Available
hair loss	4	0	GP referral for scalp problems
Acne or *Herpes simplex* reactivation	0	0	Not noted
Any pain? e.g. joint pain, headache	55	24	Care plan to monitor non-verbal clues and pain charts. Analgesia. Dental referral.
Regular medication taken at the same time each day	0	0	Not actioned.
More than 2 doses of prescribed medication missed over any period of seven days in the last month?	21	1	GP referral. Medicines changed to liquid formulations.

In adjusted analyses, more problems were found (adjusted mean difference 9.06 [95% CI 7.95–10.16]), and addressed (adjusted mean difference 3.34 [2.57–4.11]) when the Profile was used. These increases were also associated with older age and higher number of prescription medicines at baseline. Number of problems addressed varied with trial step, and was significantly lower in steps 2–5, when Profile use and other factors were accounted (Table 6).

**Table 6:** Profile Effect on selected outcomes: adjusted analyses. Notes: D = raw, observed (unadjusted) difference in the same units as the variable; β = β coefficient of profile effect or effect size in the same units as variable; OR = raw (unadjusted odds ratio); aOR = adjusted Odds Ratio, exponent of β. ADL = activities of daily living. n represents the number of data collection points.

Variable	Overall	Without profile	With profile	Profile Effect		95% CI for Adjusted Effect		ICC**	Significant covariates	P values	Adjusted effect, parameters, β or aOR (95% CI)
**Primary outcomes**				Raw	Adjusted*	Lower	Upper				
**Total Problems**									Step 1	0.007	β = 1.82 (0.51–3.13)
**Found**									Step 4	0.03	β = -1.24 (-2.36–-0.12)
Mean (sd)	11.52(6.36)	7.30(3.18)	15.81(5.90)	D = 8.51	β = 9.06	7.95	10.16	r = 0.16	Age	0.003	β = 0.16 (0.06–0.27)
[n]	[249]	[125]	[124]		(P<0.001)				Number of medicines prescribed†	0.003	β = 0.36 (0.13–0.60)
**Total Problems**									Step 2	<0.001	β = -2.08 (-3.02–1.14)
**Addressed**									Step 3	0.03	β = -1.04 (-1.96–-0.12)
Mean (sd)	7.93(4.23)	6.02(2.92)	9.86(4.48)	D = 3.84	β = 3.34	2.57	4.11	r = 0.12	Step 4	<0.001	β = -2.45 (-3.34–1.52)
[n]	[249]	[125]	[n = 124]		(P<0.001)				Step 5	<0.001	β = -1.72 (-2.67–0.77)
									Age	0.002	β = 0.14 (0.05–0.22)
									Number of medicines prescribed†	0.006	β = 0.26 (0.08–0.45)
**Secondary outcomes**											
**Mental health Medicines Reduction**											
Proportion (%)	18/208(8.7%)	3/84(3.6%)	15/124(12.1%)	OR = 3.72	aOR = 4.45(P = 0.03)	1.15	17.22	r = 0.02	Number of medicines prescribed†	0.03	aOR = 1.17 (1.01–1.35)
**MOUSEPAD**											
Mean (sd)	7.53(6.47)	7.31(6.90)	7.76(6.04)	D = 0.45	β = 0.23	-0.47	0.93	r = 0	Antipsychotics at	0.03	β = 4.22 (0.37–8.07)
[n]	[249]	[125]	[124]		(P = 0.52)				baseline		
**Bristol (ADL) Score**											
Mean (sd)	30.13(14.48)	27.81(14.52)	32.48(14.11)	D = 4.67	β = 1.37	-0.04	2.78	r = 0.21	None		
[n]	[249]	[125]	[124]		(P = 0.06)						
**Examples of nature of problems addressed**											
**Falls Action**	66/249	34/125	32/124	OR = 0.94	aOR = 0.73	0.34	1.55	r = 0.01	None		
Proportion (%)	(26.4%)	(27.0%)	(25.8%)		(P = 0.407)						
**Falls** Documented	66/249	35/125	31/124	OR = 0.86	aOR = 0.62	0.29	1.32	r = 0	none		
Proportion (%)	(26.5%)	(28.0%)	(25.0%)		(P = 0.22)						
**Pain Actions**	79/249	24/125	55/124	OR = 3.39	aOR = 3.84				Step 2	0.05	aOR = 0.33 (0.11–1.00)
Proportion(%)	(31.6%)	(19.0%)	(44.4%)		(P = 0.001)	1.78	8.30	r = 0.01	Number of medicines prescribed†	0.007	aOR = 1.23 (1.06–1.43)
**Pain Documented**	80/249	25/125	55/124	OR = 3.19	aOR = 3.45				Step 2	0.04	aOR = 0.32 (0.11–0.96)
Proportion (%)	(32.1%)	(20.0%)	(44.4%)		(P = 0.001)	1.63	7.30	r = 0	Number of medicines prescribed†	0.005	aOR = 1.23 (1.07–1.43)
**Sedation Documented**	35/249	7/125	28/124	OR = 4.92	aOR = 8.67				Age	0.02	aOR = 1.08 (1.01–1.16)
Proportion (%)	(14.1%)	(5.6%)	(22.6%)		(P<0.001)	2.90	25.86	r = 0.06			
**Before and after analyses**											
**Optician Visit**	64/82	27/41	37/41	OR = 4.796	aOR = 5.12	1.45	18.03	r = 0	None		
Proportion (%)	(78.0%)	(65.8%)	(90.2%)		(P = 0.01)						
**Dentist Visit**	35/82	4/41	31/41	OR = 28.67	aOR = 52.76	11.80	235.90	r = 0.122	None		
Proportion (%)	(42.7%)	(9.8%)	(75.6%)		(P<0.001)						

* adjusted for Step, age, number of medicines at baseline, antipsychotics, antiepileptics, antidepressants and SSRIs at baseline;

** ICC, intracluster correlation coefficient, based on raw data;

† number of medicines recorded as prescribed at baseline.

#### b. Benefits

The Profile affected the care of most, but not all, participants. We identified clinical impact and specific examples, guided by previous work[24]. Some actions related to direct intervention to manage symptoms, such as referral for GI symptoms (P20, Table 7) or increased pain relief, whilst others related to health promotion, for example changes to diet and mouth care. These items were important to address some of the nuanced effects of ADRs. The intervention increased visits to dentists and opticians. At baseline, 4 had visited dentists and 27 opticians, within the last 12 months. By the final step, this had increased to 31 and 37, in association with Profile use (aOR 52.76 [11.80–235.90] and 5.12 [1.45–18.03] respectively). The importance of this is illustrated by P2 (Table 7).

**Table 7:** Case Reports of benefits of Profile use.

Participant number	Theme	Report
P20	Investigating poor intake	A lady in her mid 90s with a diagnosis of mild to moderate dementia was prescribed: ranitidine, aspirin, furosemide, simvastatin, levothyroxine, zopiclone, ramipril, sodium valproate as Epilim^®^. On the second use of the Profile, abdominal pain was identified as a possible cause of irregular dietary/ food intake. This was followed up with the GP, and the participant was investigated for gastro-intestinal ulceration.
P2	Investigating poor intake	A gentleman of in his mid 70s with a diagnosis of Alzheimer’s disease, ischaemic heart disease, and arthritis had no record of problems with dietary intake or dentists’ visits before the Profile was introduced. On first use, the Profile identified dental problems, poor intake and missed or unfinished meals. A dental appointment was then arranged, and the dentist needed to extract a decayed tooth. On subsequent assessments, meals were no longer missed, fruit and milk intake had improved and sugary drinks were no longer used.
P19	Recognising ADRs	A lady in her late 80s with a diagnosis of Alzheimer’s disease was prescribed mirtazapine, lactulose, senna, diazepam, aspirin, omeprazole, folic acid, alendronic acid, vitamin D supplements as Calcichew D3 forte^®^, simvastatin, and risperidone as needed. Initial monitoring (Step 4) indicated several problems including: cognitive decline, violence, aggression, agitation, hallucinations, risk of falls, problems with dentures, and pain. The nursing team were proactive in discontinuing risperidone, and violence, aggression, agitation, hallucinations, falls, denture problems and pain resolved. One month later, the potential problem of falls was incorporated into the personalised care plans for the first time, triggering discontinuation of diazepam.
P16	Recognising ADRs	A lady in her late 80s, diagnosed with dementia was prescribed hyoscine as needed, oxazepam, carbamazepine, omeprazole, levomepromazine, senna, magnesium hydroxide, and paracetamol. At first administration of the Profile (Step 3), she was noted to be aggressive, restless, confused, sedated and agitated. In accordance with Profile guidelines, hyoscine had been discontinued by Step 4. By the end of the study, aggression, restlessness and sedation were no longer recorded as problematic. The potential for falls was recorded on the Profile, but not elsewhere in the records.

Eleven participants reported falls in the first step of data collection, and six in the final step. Profile use did not alter reporting and addressing falls (Table 6), but more care plans were put in place to address problems with balance (Table 5).

Nurses were more likely to document and treat pain when using the Profile. At baseline, 11/41 participants had their pain status documented; this increased to 40/41 in the last step. Fewer participants reported being in pain: 9 and 4 respectively. Without the Profile 1/41 participants received new analgesia; with the Profile this increased to 9/41, and ranged from transdermal fentanyl to liquid paracetamol. Use of the Profile increased pain-related documentation and actions (aOR 3.45 [1.63–7.30] and 3.84 [1.78–8.30] and 1.80–7.78) (Table 6). Similar large increases in actions taken were seen for problems with balance, cognitive decline, irritability, mood fluctuations, behaviour problems, restlessness, confusion, apathy, hallucinations, sedation, headache, emesis, missed meals, fluid intake, vision problems, and missed medicines. There was little change in actions for weight and girth combined, aggression, falls, and incontinence. Other problems were less frequent (Table 5).

The Profile allowed relatively unusual problems to be identified and addressed. Examples included: a new hearing problem (P9), which resolved on ear syringing; nausea (P12), which resolved when the daily dose of lactulose was divided; difficulties with urination which triggered a prescription for tamsulosin (P33); identification of gastro-intestinal ulceration (P20, Table 7).

#### c. Prescription changes

Neither medication reviews nor documentation of ADRs were located in patients’ documentation. Introduction of the Profile did not influence the total numbers of medicines prescribed, due in part to the increase in analgesia, above, and there was a small increase over time (Table 8). The baseline differences between the sites did not reach statistical significance. Although there were no overall changes in total medicines prescribed, the numbers of participants with any change increased throughout the trial (Table 9).

**Table 8:** Number of prescribed medicines in each step for each site. Bold text indicates roll-out of medicines’ monitoring and Profile administration 1 month before these data collection points (occasions when researchers extracted data from participants’ records). n = number of service users in the site. One participant from site 4 passed away between steps 3 & 4. One participant from site 5 was hospitalised between steps 3 & 4. Participants joined site 5 at steps 2 and 3. Problems explored are listed in Table 5 and on the Profile, S1 Appendix. Fuller versions of these tables, including medians and 25^th^ = 75^th^ centiles are in S3 table.

	Number of prescribed medicines					
Site	Step 1.	Step 2.	Step 3.	Step 4.	Step 5.	Step 6.
**5:** Sum (n)	85 (8)	96 (9)	109 (10)	105 (9)	108 (9)	**115 (9)**
Mean [SD]	10.63[3.07]	10.67 [3.16]	10.90 [3.28]	11.67 [2.92]	12.00 [3.12]	**12.78[40.6]**
Full range	7–15	7–16	6–16	6–15	7–16	**6–19**
**4:** Sum (n)	91 (10)	93 (10)	18 (10)	92 (9)	**98 (9)**	**101 (9)**
Mean [SD]	9.10 [4.23]	9.30 [4.24]	9.60 [3.95]	10.22 [4.30]	**10.89 [4.83]**	**11.22 [4.06]**
Full range	4–18	4–18	5–18	5–19	**6–21**	**7–18**
**3:** Sum (n)	48 (5)	50 (5)	49 (5)	**50 (5)**	**49 (5)**	**49 (5)**
Mean [SD]	9.60 [1.14]	10.00 [2.00]	9.80 [1.79]	**10.00 [1.23]**	**9.80 [1.30]**	**9.80 [1.30]**
Full range	8–11	8–12	8–12	**8–11**	**8–11**	**8–11**
**2:** Sum (n)	89 (8)	89 (8)	**89 (8)**	**88 (8)**	**85 (8)**	**89 (8)**
Mean [SD]	11.13 [3.91]	11.13 [4.29]	**11.13 [3.56]**	**11.00 [3.21]**	**10.63 [3.29]**	**11.13 [3.56]**
Full range	6–19	6–20	**6–18**	**6–17**	**6–17**	**6–18**
**1:** Sum (n)	68 (10)	**72 (10)**	**72 (10)**	**71 (10)**	**70 (10)**	**84 (10)**
Mean [SD]	6.80 [3.36]	**7.20 [3.33]**	**7.20 [3.49]**	**7.10 [3.35]**	**7.00 [3.50]**	**8.40 [3.98]**
Full range	2–13	**2–13**	**2–13**	**2–13**	**2–14**	**2–14**

**Table 9:** Numbers of participants with any change made to any of their prescribed medications at each step in each site. Bold text indicates roll-out of medicines’ monitoring and Profile administration 1 month before these data collection points (occasions when researchers extracted data from participants’ records). n = number of service users in the site. One participant from site 4 passed away between steps 3 & 4. One participant from site 5 was hospitalised between steps 3 & 4. Participants joined site 5 at steps 2 and 3. Problems explored are listed in Table 5 and on the Profile, S1 Appendix. Fuller versions of these tables, including medians and 25^th^ = 75^th^ centiles are in S3 Table.

Site	Number with changes to prescribed medicines					
	Step 1.	Step 2.	Step 3.	Step 4.	Step 5.	Step 6.
**5:** n/N (%)	NA	1/9 (11%)	1/10 (10%)	7/9 (78%)	7/9 (78%)	**6/9 (67%)**
**4:** n/N (%)	NA	3/10 (30%)	5/10 (50%)	6/9 (67%)	**7/9 (78%)**	**8/9 (89%)**
**3:** n/N (%)	NA	1/5 (20%)	1/5 (20%)	**3/5 (60%)**	**1/5 (20%)**	**4/5 (80%)**
**2:** n/N (%)	NA	2/8(25%)	**5/8 (63%)**	**6/8 (75%)**	**4/8 (50%)**	**5/8 (63%)**
**1:** n/N (%)	NA	**1/10 (10%)**	**4/10 (40%)**	**3/10 (30%)**	**4/10 (40%)**	**6/10 (60%)**

During the trial there were 18 reductions in mental health medicines affecting 12 service users: 10 while using the Profile, 1 without the Profile, and 1 both with and without. (Table 7 contains two examples). Reduction in participants’ mental health medicines was associated with Profile use (aOR 4.45 [1.15–17.22]) and the number of prescription medicines at baseline (aOR 1.17 [1.01–1.35} per medicine) (Table 6).

At baseline, 3/41 participants had sedation levels documented, and recording was complete by the final step. Three participants had their sedatives reduced (trazodone, lorazepam, risperidone).

#### d. Functional status and dementia severity

By the end of the study, Bristol ADL scores and the MOUSEPAD measure of dementia behavioural and psychological symptoms had deteriorated slightly. Changes were not statistically significantly associated with Profile use: Deterioration in dementia severity was associated with antipsychotic prescription at baseline (Table 6).

### 2. Potential Costs

The direct cost of Profile administration was typically 15–30 minutes time from band 5 nurses, the starting point for newly registered qualified nurses, an estimated £20.50 ($31.5, €28.2), based on salary costs of £41 per hour[69]. Some nurses needed an hour to read through the guidelines carefully before first administration, and administration times ranged 10–60 minutes, costing £7–41 ($10.8–63.0, €9.6–56.4). Administration times were shorter when nurses had familiarised themselves with the appended guidelines. Addressing problems or care deficits identified by the Profile entailed indirect costs: for example, referrals and visits to opticians and dentist increased in association with the intervention. A visit to the community dentist is estimated to cost £115 ($176.7, €158.2) and an optician £21 ($32.3, €28.9)[70]. Some prescribing changed, but no changes in high cost medicines were observed.

Potential savings were identified. Improved vision, following opticians’ input, combined with reduction of medicines such as diazepam and antipsychotics, which are associated with dizziness, falls and fractures[60,71], could lead to savings by reducing falls. The costs of pain, sedation, poor oral hygiene and poor eyesight, and their amelioration, are hard to quantify, but may affect Health Related Quality of Life.

### 3. Harms

Implementation of the WWADR Profile was neither observed nor reported to be directly harmful, and Bristol ADL and MOUSEPAD scores did not deteriorate significantly. The research entailed no more than minimal risk, defined as clinical examination and record review[66]: the Profile has been freely available in the public domain since 2004[42], and all questions and observations are of a nature that should be pursued under routine care. The time taken identifying and responding to problems may have distracted nurses from other tasks, but prioritisation of actions was at the discretion of the multidisciplinary team. Medicines were changed in response to needs identified. Neither participants nor their families reported negatively on the amount of time spent in completing the WWADR Profile. At recruitment, pressure of existing work was cited by nurse managers, acting as gatekeepers, as their reason for non-participation; without further information from sites not involved, it is difficult to explore how this might relate to the 30 minutes needed to administer the Profile.

## Discussion

To our knowledge, no previous trials have tested nurse-led medicines’ monitoring interventions in a cohort stepped wedge cluster RCT. Our intervention increased the numbers of problems identified and addressed, improved pain management, and reduced prescriptions of mental health medicines. We identified potential to augment quality and safety of care and reduce costs.

### Strengths and Limitations

**Generalisability** of findings is supported by consistency across the majority of trial sites, low attrition, and with earlier work[22–25], with the *caveats* that those too ill to undergo measurements of vital signs were excluded, and all sites had pre-existing contact with the University and were in an area of the European Union (EU) where GDP is 75% below the community average[72]. The site (3) with the lowest recruitment and oldest participants (Table 1) addressed fewer problems but changed more medicines than others (Tables 4 and 9), and completion of some items relied on nurses’ intimate knowledge of participants who lacked the cognitive ability to understand questions, suggesting that clinical gains may vary between settings. International transferability of findings rests with readers’ interpretations. Prescribing patterns, including number of medicines prescribed (median 9, range 2–19) reflects previous reports[73]. The 50% recruitment rate for sites warrants cautious generalisation of findings to care homes feeling too pressurised to participate in research. Any association with nurses’ self-reported lack of confidence in medicines’ management[74] or the requirement for the Profile to be administered is uncertain. Volunteer[75] and selection[76] bias within sites was minimised by the high participation rate. This was attributed to the REC’s support for witnessed verbal assent to participation, in accordance with the Ottawa statement[66], to avoid overburdening participants’ relatives, many of whom were frail and elderly[42].

Nursing work that is unrecorded is regarded as incomplete or ‘not done’[77]. However, some observations and actions, particularly vital signs (Table 2), were **incompletely documented**, despite the known risk of hypotension associated with antipsychotics[78] and antidepressants[34]., and communications’ systems did not facilitate transfer of laboratory results. However, care homes’ records are regularly inspected by licensing authorities (CSSIW), ensuring that all medicines administered are fully and accurately recorded. We think it unlikely that social desirability responses or the Hawthorne[79] effect would have affected nurses’ completion of documentation unevenly across trial sites and steps.

We acknowledge the potential for “**exposure suspicion bias**” [80:55] to increase the numbers of problems recorded. Conversely, some problems, such as poor balance or gait may be overlooked or accepted as integral to participants’ conditions. Such “entrapment by prior expectations” [81:38] may affect profiling of ADRs[42], but is likely to have been consistent throughout the trial. We anticipated some fluctuations in recording over time, because signs and symptoms change. For example, bruises appear, eating patterns alter, and if people become dehydrated, due to changes in temperature, intake or infection, drug elimination decreases, worsening any ADRs.

**Blinding** during administration, data capture and outcome assessment in non-pharmacological[82], cluster[48] or pragmatic trials may be impossible[83], increasing risk of bias[84], in line with observers’ expectations[85]. Nurses and some, but not all, participants were aware of administration of the Profile. The presence of the Profiles in the notes precluded blinding during data collection, and the obvious increase in problem detection with the Profile (Table 3) was apparent to data analysts. Detection bias of outcomes was minimised, but not removed, by a pre-arranged data collection template and the low subjectivity of outcome data extracted from standardised documentation, such as medicines’ administration records or nutrition assessments[86]. Systematic review indicates that some 3% unblinded assessments are likely to be misclassified[87], which would not materially affect interpretation of our findings.

**Contamination** by subconscious transfer of aspects of an intervention to controls may dilute its effects[88]. Introduction of the study before implementation may have sensitised staff to medicines’ management, and medicine changes increased before use of the Profile in sites 4 & 5 (Table 6). The sites were unlikely to have discussed the trial as they were geographically dispersed and all nurses worked at only one site.

**Global outcome measures** may be better suited to population comparisons than clinical trials, as they appear to be insufficiently sensitive to prescription changes[19,89], and subtle but important improvements, such as increased oral hygiene and analgesia. Therefore, interventions to optimize prescribing, identification and resolution of medication-related problems or pain may not influence scores on global outcome measures[19,57,90–94]. While nurse-led and pharmacist-led interventions improve prescribing[95,96], mainly in patients not receiving specialist care[27], they may not change global outcome measures[19,41,89,97–99]. Although care improved, for example by reducing prescribing of sedatives and increasing administration of analgesia, global measures did not. Scores for activity of daily living (Bristol ADL) and psychopathology (MOUSEPAD) remained unchanged; however, participants were suffering from dementia, which would normally deteriorate over 5–6 months. The ambivalence between auditable processes of care, such as medication administration[3,4] and global outcomes, such as levels of functioning, reflects the inconsistency in the links between the processes and outcomes of care.

The risks of **multiple testing** were balanced against the benefits of formalising the significance of observed differences. We did not apply statistical tests to all items that could have been actioned, and the observed differences (Table 5) should be considered together with the formal analyses presented in Table 6.

### Addressing the ADR Problem

#### 1. Clinical gains

Dose titration for people with dementia is challenging: medicating the neuropsychiatric symptoms of dementia is of uncertain benefit[61,100–103], and antipsychotics are not recommended unless other measures have failed and then only for short-term administration[104]. Routine administration of a structured, standardised instrument was a low risk strategy to facilitate medication titration and compensate for any under-ascertainment of ADRs or under-treatment due to patients’ impaired communication. It was also successful in enhancing care planning to address nursing needs, such as poor fluid intake, missed meals and behaviour problems[3,4] (Tables 2 and 5).

Some 15% of older adults in the UK are prescribed at least 1 potentially inappropriate medicine[105], and medicines’ management is crucial to the optimisation of long-term conditions and avoidance of harm. However, many ADRs are subtle and ill-defined, and signs and symptoms, such as cognitive impairment[61] or impaired continence[106], may resemble pre-existing conditions or ageing or dehydration. Their aetiology is often multifactorial or uncertain[7]: for example, cognitive impairment may reduce fluid intake[3,4,107], exacerbating urinary symptoms and constipation[108], which are also ADRs to antidepressants, benzodiazepines and antipsychotics[52,34]. Determination of aetiology for each problem or assessment of appropriateness of prescribing is outside the remit of the Profile. For example: xerostomia warrants nursing or dental interventions whether due to dehydration, antipsychotics, SSRIs, AEDs, benzodiazepines or anti-muscarinics; similarly, confusion should be brought to the attention of prescribers whether due to dementia, or caused or exacerbated by cardiovascular agents, antipsychotics, SSRIs, AEDs, benzodiazepines or anti-muscarinics[34,52,53,61]. Rather, by addressing the diverse problems vulnerable to exacerbation by prescribed medicines[52,109], the Profile works towards incremental optimisation of health. In view of the paucity of the evidence base on potential adverse drug reactions[110–112], to optimise clinical gain, the Profile makes a thorough and detailed check for potential medication-related harms, risking over-ascertainment, rather than overlooking potentially treatable problems of equivocal aetiology. Interpretation of such inclusivity rests with readers. The diversity of problems identified and addressed suggests there may be some merit in compiling a single Profile of problems ahead of physician or pharmacist review.

Previously, introduction of nurse-led medicines’ monitoring expedited the recognition and treatment of unsuspected problems, such as orthostatic hypotension, coupled beats, hypertension, constipation, and inadequate diet[22,23], need for medication review, immunisations[25], suboptimal oral care, pain and sedation[24]. Including a question on pain in a comprehensive profile improved pain management, as did administration of purposive instruments[57,113]. Similarly, pharmacist-led medicines’ review yielded greater clinical gains by treating previously unrecognised symptoms than discontinuing inappropriate medicines[114].

#### 2. Costs

Additional healthcare needs emanating from medicines’ mismanagement cost the USA $213bn (8% of total healthcare spend) in 2012, mainly due to an additional 10m hospitalisations[115]. Inappropriate prescribing to those aged ≥70 in Northern Ireland in 2009/2010 cost €6,098,419, 5.38% of overall pharmaceutical expenditure[116]. Pharmacist review made a non-significant difference to nursing home residents’ healthcare costs[117], and was ineffective in older people in the community[90]. Our Profile was easy to administer and addressed the problems of incomplete documentation (Table 1), non-adherence (Tables 2 and 5), errors, inappropriate prescribing (Tables 6 and 9) and mismanagement cheaply (~£20). The Profile increased nurses’ workloads: not only was time needed for completion with participants, but also for contacting prescribers, reviewing care plans, arranging GP, dental and optician appointments, to the benefit of participants. Whilst the results of the monitoring affected prescribing and referrals, this was within the context of standard NHS care, and these additional NHS costs were not direct results of the monitoring. We did not have the resources to estimate the costs or savings for each participant.

#### 3. Harms

Medicines’ monitoring and review are reported not to cause harm[19,92,93], and the additional care plans and referrals reported had no risk attached (Table 5). Profiles should neither duplicate existing documentation nor increase professionals’ bureaucratic burden[118], but there is merit in a single instrument to identify, communicate and address problems. Where nurses undertake tasks outside traditional nursing roles, time and educational preparation[74] are perceived barriers. Any change needs to be seen as beneficial, resourced and achievable[6]. To allay concerns regarding the necessary investment in time and learning, the clinical effectiveness of ADR profiles in identifying and ameliorating the burdens of treatment should be demonstrated[119].

#### A Strategy worth exploring

**Fig 2:**
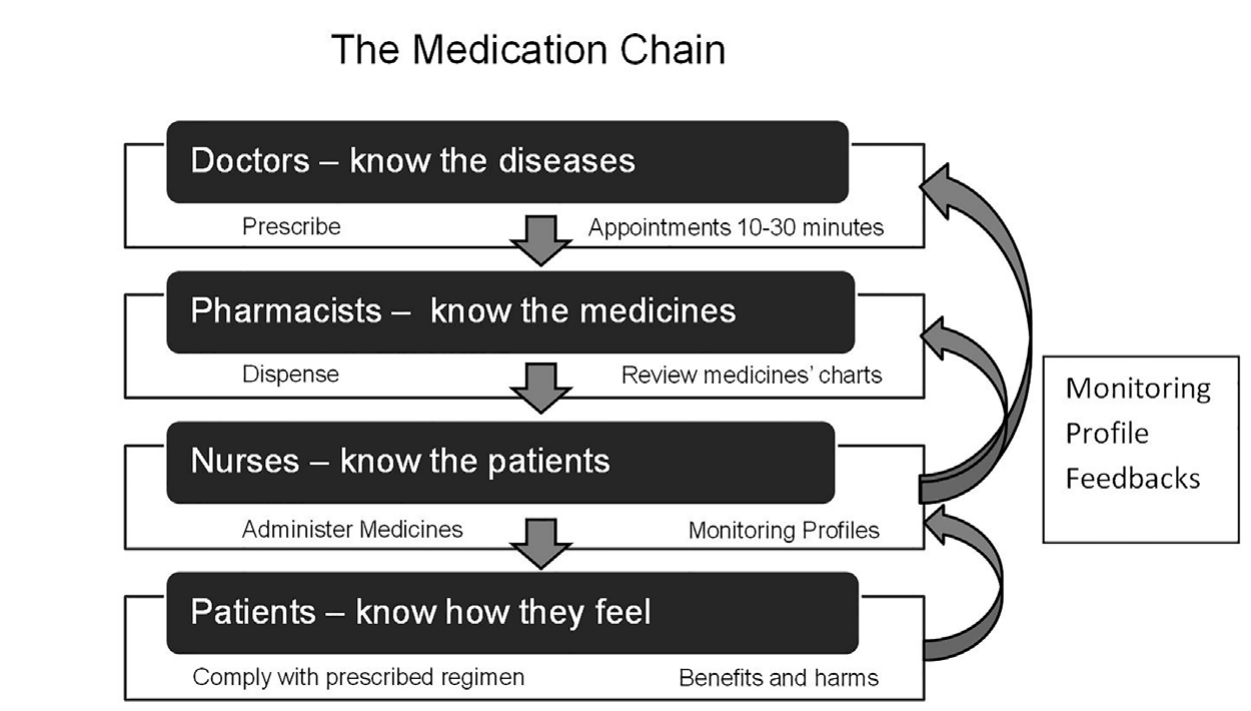
The medication chain.

Neither guidelines[15,35,43] nor literature reviews[6] offer consensus or structures for identifying and addressing medicine-related harm and adverse events. Further research into increased nursing vigilance and improved systems for actively monitoring patients for known adverse effects of prescribed medicines is warranted[20,120–122], and monitoring profiles are ideal[123] (Fig 2). Profiles do not replace clinical knowledge and experience: they juxtapose information on signs and symptoms with therapeutic regimens in a succinct, formal assessment, and suggest solutions to problems potentially related to prescribed medicines[124]. Our Profile augments professionals’ and patients’ awareness of ADRs[125], and would mitigate public bodies’ concerns over iatrogenesis[1,2,112,126] and under-reporting of ADRs[127,128]. Adoption would expedite: pain management[662,124], proactive and prompt sharing of complete prescribing information during patient transfer[35:77], and recommendations from inquiries into health care failings. These include: enhanced transparency; inter-disciplinary teamwork[4]; informing patients of their medicines’ adverse effects[3:418]; easily accessible and systematic recording of routine observations[3:129]; frequent checking to minimize medication errors[3:1613]; a “zero tolerance” approach to the improper administration of sedation and medicines[3:111,4:34]; comprehensive education initiatives in medication management[4:R8]; monitoring food and fluid intake[3:111,4:R4].

### Conclusion

Nurse-led medicines’ monitoring improved some aspects of care, including prescribing and pain management, and focused attention on participants’ reports of adverse events[99,129]. The intervention is feasible[24], low cost, low risk and, when operationalized, convenient for service users and professionals, and offers potential for cost savings and increased quality and safety of care. However, larger, multicentre trials are needed to examine long-term effects of structured medicines’ monitoring on clinical outcomes[130], nurses’ workloads, and bridging the gap between patients and prescribers (Fig 2)[20,120–122].

## Supporting Information

S1 Appendix West Wales Adverse Drug Reaction profile for medicines in mental health v. IX. (DOCX)

S2 Appendix The West Wales Adverse Drug Reaction (WWADR) Profile for Mental Health Medicines: template for intervention description and replication (TIDieR) checklist. (DOCX)

S1 CONSORT Checklist CONSORT 2010 checklist of information to include when reporting a cluster randomised trial (Campbell et al 2012). (DOCX)

S1 Protocol Protocol for the study. (PDF)

S1 Table Total number of problems found per participant at each step and in each site. This is the S1 table legend reporting descriptive data in full.
(DOCX)

S2 Table Total number of actions taken per participant at each step in each site. This is the S2 table legend reporting descriptive data in full.
(DOCX)

S3 Table Number of prescribed medicines in each step for each site. This is the S3 table legend reporting descriptive data in full.
(DOCX)

## Abbreviations

ADLs: Activities of daily living
AIMS: Abnormal Involuntary Movements Scale
BMI: Body mass index
BNF: British National Formulary
BP: Blood pressure
CNS: Central nervous system
CPK: Creatine phosphokinase
ECG: Electrocardiograph
FBC: Full blood count
GP: General practitioner
LFTs: Liver function tests
RBC: Red blood cell
TFTs: Thyroid function tests
UTI: Urinary tract infection

## References

[1] National Patient Safety Agency (NPSA). Safety in doses: medication safety incidents in the NHS National Patient Safety Agency, London 2007.

[2] Committee of Public Accounts. A Safer Place for Patients: Learning to Improve Patient Safety. London, UK: The Stationery Office, 2006.

[3] Francis R. Report of the Mid Staffordshire NHS Foundation Trust Public Inquiry: Executive Summary. (Online). Available: http://www.midstaffspublicinquiry.com/report Accessed 4 June 2015.

[4] Andrews J, Butler M. Trusted to Care An independent Review of the Princess of Wales Hospital and Neath Port Talbot Hospital at Abertawe Bro Morgannwg University Health Board People, Dementia Services Development Centre, the People Organisation. 2014. Available: http://wales.gov.uk/docs/dhss/publications/140512trustedtocareen.pdf Accessed 4 June 2015.

[5] Jordan S, Kyriacos U. Medicines’ Management: a public health problem on nursing’s agenda. Journal of Nursing Management. 2014; 22(3):271–5 10.1111/jonm.12238 24754749

[6] Gabe M, Murphy F, Davies G, Davies M, Johnstone L, Jordan S. Adverse events and nurse-led medication monitoring. Journal of Nursing Management 2011; 19, 377–392 21507109 10.1111/j.1365-2834.2011.01204.x

[7] International Conference on Harmonisation (ICH) ICH Harmonised Tripartite Guideline for Good Clinical Practice. Institute of Clinical Research, Marlow, Buckinghamshire. 1996. Available: http://www.ich.org/fileadmin/Public_Web_Site/ICH_Products/Guidelines/Efficacy/E6/E6_R1_Guideline.pdf. Accessed 4 June 2015.

[8] Davies EC, Green CF, Mottram DR, Rowe PH, Pirmohamed M. Emergency re-admissions to hospital due to adverse drug reactions within 1 year of the index admission. British Journal of Clinical Pharmacology. 2010; 70 (5), 749–55. 10.1111/j.1365-2125.2010.03751.x 21039769 PMC2997315

[9] Pirmohamed M, James S, Meakin S, Green C, Scott AK, Walley TJ, et al Adverse drug reactions as cause of admission to hospital: prospective analysis of 18 820 patients. BMJ. 2004; 329(7456), 15–19. 15231615 10.1136/bmj.329.7456.15 PMC443443

[10] Marcum ZA, Amuan ME, Hanlon JT, Aspinall SL, Handler SM, Ruby CM, et al Prevalence of unplanned hospitalizations caused by adverse drug reactions in older veterans. Journal of the American Geriatric Society. 2012; 60 (1), 34–41. 10.1111/j.1532-5415.2011.03772.x PMC3258324 22150441

[11] Howard R, Avery A, Slavenburg S, Royal S, Pipe G, Lucassen P, et al Which drugs cause preventable admissions to hospital? A systematic review. Bristish Journal of Clinical Pharmacology. 2007; 63 (2), 136–47. 10.1111/j.1365-2125.2006.02698.x PMC2000562 16803468

[12] Wu TY, Jen MH, Bottle A, Molokhia M, Aylin P, Bell D, et al Ten-year trends in hospital admissions for adverse drug reactions in England 1999–2009. Journal of the Royal Society of Medicine.2010; 103 (6), 239–50. 10.1258/jrsm.2010.100113 20513902 PMC2878823

[13] Frontier Economic. Exploring the costs of unsafe care in the NHS: a report prepared for the department of health, Frontier Economics, London 2014 Available: http://www.frontier-economics.com/documents/2014/10/exploring-the-costs-of-unsafe-care-in-the-nhs-frontier-report-2-2-2-2.pdf. Accessed 4 June 2015.

[14] Sultana J, Cutroneo P, Trifirò G. Clinical and economic burden of adverse drug reactions. Journal of Pharmacology and Pharmacotherapeutics. 2013; 4 (Suppl1), S73–S77. 24347988 10.4103/0976-500X.120957 PMC3853675

[15] NICE Medicines & Prescribing Centre. Medicines optimisation: the safe and effective use of medicines to enable the best possible outcomes NICE guideline 5. NICE, London 2015 Available: http://www.nice.org.uk/guidance/ng5/evidence/full-guideline-6775454 Accessed 4 June 2015.

[16] Hakkarainen KM, Andersson Sundell K, Petzold M, Hägg S. Prevalence and perceived preventability of self-reported adverse drug events-a population-based survey of 7099 adults. PLoS ONE. 2013; 8 (9), e73166 10.1371/journal.pone.0073166 24023828 PMC3762841

[17] Forster AJ, Murff HJ, Peterson JF, Gandhi TJ, Bates TW. Adverse drug events occurring following hospital discharge. Journal of General Internal Medicine 2005; 20(4) pp.317–323. 15857487 10.1111/j.1525-1497.2005.30390.x PMC1490089

[18] Gurwitz J, Field T, Judge J, Rochon P, Harrold LR, Cadoret C, et al The incidence of adverse drug events in two large academic long-term care facilities. The American Journal of Medicine 2005; 118, 251–8 15745723 10.1016/j.amjmed.2004.09.018

[19] Alldred DP, Raynor DK, Hughes C, Barber N, Chen TF, Spoor P. Interventions to optimise prescribing for older people in care homes. Cochrane Database of Systematic Reviews 2013; Issue 2 Art. No.: CD009095 10.1002/14651858.CD009095.pub2 23450597

[20] Steinman MA, Handler SM, Schiff GD, Covinsky KE. Beyond the prescription: medication monitoring and adverse drug events in older adults. 2011; 59(8) pp. 1513–20. 10.1111/j.1532-5415.2011.03500.x PMC3940362 21797831

[21] Brenner S, Detz A, López A, Horton C, Sarkar U. Signal and noise: applying a laboratory trigger tool to identify adverse drug events among primary care patients. BMJ Qual Saf. 2012; 21(8):670–5. 10.1136/bmjqs-2011-000643 22626736 PMC3402751

[22] Jordan S. Managing Adverse Drug Reactions: An Orphan Task. Developing nurse-administered evaluation checklists. Journal of Advanced Nursing 2002; 38:5: 437–448. 10.1046/j.1365-2648.2002.02205.x 12028277

[23] Jordan S, Tunnicliffe C, Sykes A. Minimising Side Effects: The clinical impact of nurse-administered ‘side effects’ checklists. Journal of Advanced Nursing 2002; 37;2:155–65. 11851783 10.1046/j.1365-2648.2002.02064.x

[24] Jordan S, Gabe M, Newson L, Snelgrove S, Panes G, Picek A, et al Medication Monitoring for People with Dementia in Care Homes: the Feasibility and Clinical Impact of Nurse-led monitoring. The Scientific World Journal, vol. 2014, Article ID 843621, 11 pages, 10.1155/2014/843621 PMC3951004 24707218

[25] Gabe ME, Murphy F, Davies GA, Russell IT, Jordan S. Medication monitoring in a nurse-led respiratory outpatient clinic: pragmatic randomised trial of the West Wales Adverse Drug Reaction Profile. PLOS One 2014;9(5) e96682 10.1371/journal.pone.0096682 24798210 PMC4010491

[26] Banerjee S. The use of antipsychotic medication for people with dementia: time for action. A report for the Minister of State for Care Services. An Independent |Report commissioned for the Department of Health, London 2009. Available: http://www.dh.gov.uk/prod_consum_dh/groups/dh_digitalassets/documents/digitalasset/dh_108302.pdf. Accessed 4 June 2015.

[27] Child A, Clarke A, Fox C, Maidment I. A pharmacy led program to review anti-psychotic prescribing for people with dementia. BMC Psychiatry. 2012;12:155 10.1186/1471-244X-12-155 23006528 PMC3472196

[28] Alessi-Severini S, Dahl M, Schultz J, Metge C, Raymond C. Prescribing of psychotropic medications to the elderly population of a Canadian province: a retrospective study using administrative databases. PeerJ, 2013;1, e168 10.7717/peerj.168 24109553 PMC3792174

[29] Harris T, Carey IM, Shah SM, DeWilde S, Cook DG. Antidepressant prescribing in older primary care patients in community and care home settings in England and Wales. J Am Med Dir Assoc. 2012;13(1):41–7. 10.1016/j.jamda.2010.09.005 21450211

[30] Stefan H. Epilepsy in the elderly: facts and challenges. Acta Neurol Scand. 2011; 124(4):223–37. 10.1111/j.1600-0404.2010.01464.x 21143593

[31] Laroche ML, Perault-Pochat MC, Ingrand I, Merle L, Kreft-Jais C, Castot-Villepelet A, et al Adverse drug reactions in patients with Alzheimer's disease and related dementia in France: a national multicentre cross-sectional study. Pharmacoepidemiol Drug Saf. 2013 9;22(9):952–60. 10.1002/pds.3471 Epub 2013 Jun 24. 23794320

[32] Gallini A, Andrieu S, Donohue JM, Oumouhou N, Lapeyre-Mestre M, Gardette V. Trends in use of antipsychotics in elderly patients with dementia: Impact of national safety warnings. Eur Neuropsychopharmacol. 2014 1;24(1):95–104. 10.1016/j.euroneuro.2013.09.003 PMC4094113 24126116

[33] FDA. Information for Healthcare Professionals: Conventional Antipsychotics. 2008. Available: http://www.fda.gov/Drugs/DrugSafety/PostmarketDrugSafetyInformationforPatientsandProviders/ucm124830.htm. Accessed 4 June 2015.

[34] British National Formulary. BNF number 69. London, UK: British Medical Association and the Royal Pharmaceutical Society of Great Britain 2015

[35] NICE. Psychosis and Schizophrenia in adults: treatment and interventions management of schizophrenia in primary and secondary care (update). Clinical Practice Guideline no. 178. Centre for Clinical Practice. Commissioned by NICE. 2014. Available: http://www.nice.org.uk/guidance/cg178/resources/cg178-psychosis-and-schizophrenia-in-adults-full-guideline3. Accessed 4 June 2015.

[36] Raynor DK, Blenkinsopp A, Knapp P, Grime J, Nicolson DJ, Pollock K, et al A systematic review of quantitative and qualitative research on the role and effectiveness of written information available to patients about individual medicines. Health Technol Assess. 2007;11(5):iii, 1–160. 17280623 10.3310/hta11050

[37] Nicolson D, Knapp P, Raynor DK, Spoor P. Written information about individual medicines for consumers. Cochrane Database Syst Rev. 2009;(2):CD002104 10.1002/14651858.CD002104.pub3 19370575 PMC6483695

[38] Grant AM, Guthrie B, Dreischulte T. Developing a complex intervention to improve prescribing safety in primary care: mixed methods feasibility and optimisation pilot study. BMJ Open. 2014;4 (1), e004153 10.1136/bmjopen-2013-004153 24448848 PMC3902335

[39] RESPECT Trial Team. Effectiveness of shared pharmaceutical care for older patients: RESPECT trial findings. *Br J Gen Pract* 2009; 10.3399/bjgp09X473295 (abridged text, in print: *Br J Gen Pract* 2010; 59: 14–20). PMC2801801 19995493

[40] Forsetlund L, Eike MC, Gjerberg E, Vist GE. Effect of interventions to reduce potentially inappropriate use of drugs in nursing homes: a systematic review of randomised controlled trials. BMC Geriatr. 2011;11:16 10.1186/1471-2318-11-16 21496345 PMC3108292

[41] Holland R, Desborough J, Goodyer L, Hall S, Wright D, Loke YK. Does pharmacist-led medication review help to reduce hospital admissions and deaths in older people? A systematic review and meta-analysis. Br J Clin Pharmacol. 2008;65(3):303–16. 18093253 10.1111/j.1365-2125.2007.03071.x PMC2291244

[42] Jordan S, Knight J, Pointon D. Monitoring adverse drug reactions: scales, profiles, and checklists. International nursing review, 2004;51(4), 208–221. 15530161 10.1111/j.1466-7657.2004.00251.x

[43] Bakerjian D. CMS national partnership to improve dementia care in nursing homes Geriatric Nursing. 2014;35 (1), 77–79

[44] Woertman W, de Hoop E, Moerbeek M, Zuidema SU, Gerritsen DL, Teerenstra S. Stepped wedge designs could reduce the required sample size in cluster randomized trials. Journal of Clinical Epidemiology. 2013;66(7) 752–758. 10.1016/j.jclinepi.2013.01.009 23523551

[45] Brown CA, Lilford RJ. The stepped wedge trial design: a systematic review. BMC Medical Research Methodology 2006;6:54 17092344 10.1186/1471-2288-6-54 PMC1636652

[46] Keriel-Gascou M, Buchet-Poyau K, Duclos A, Rabilloud M, Figon S, Dubois JP, et al Evaluation of an interactive program for preventing adverse drug events in primary care: study protocol of the InPAct cluster randomised stepped wedge trial. Implementation Science. 2013;8:69 10.1186/1748-5908-8-69 23782470 PMC3689059

[47] Mdege ND, Man MS, Taylor Nee Brown CA, Torgerson DJ. Systematic review of stepped wedge cluster randomized trials shows that design is particularly used to evaluate interventions during routine implementation. J Clin Epidemiol. 2011;64(9):936–48. 10.1016/j.jclinepi.2010.12.003 21411284

[48] Campbell MK, Piaggio G, Elbourne DR, Altman DG, CONSORT Group. Consort 2010 statement: extension to cluster randomised trials. BMJ. 2012;345:e5661 10.1136/bmj.e5661 22951546

[49] National Quality Forum MCC Measurement Framework Final Report, Washington DC. 2012. Available: http://www.qualityforum.org/Publications/2012/05/MCC_Measurement_Framework_Final_Report.aspx. Accessed 4 June 2015.

[50] Jordan S, Hardy B, Coleman M. Medication management: an exploratory study into the role of Community Mental Health Nurses. Journal of Advanced Nursing. 1999:29:5: 1068–81 10320489 10.1046/j.1365-2648.1999.01002.x

[51] Jordan S, Jones R, Sargeant M. Adverse drug reactions: managing the risk. Journal of Nursing Management. 2009;17; 175–84 10.1111/j.1365-2834.2009.00977.x 19416420

[52] Jordan S. The Prescription Drug Guide for Nurses. Open University Press, McGraw-Hill, Maidenhead 2008.

[53] Taylor D, Paton C, Kapur S. The Maudsley Prescribing Guidelines, 11th Edition, Wiley Blackwell, Oxford 2012.

[54] Uitenbroek DG. "SISA-Binomial" (Simple Interactive Statistical Analysis). 1997 SISA. Available: http://home.clara.net/sisa/sampshlp.htm. Accessed 4 June 2015.

[55] Killip S, Mahfoud Z, Pearce K. What Is an Intracluster Correlation Coeffi cient? Crucial Concepts for Primary Care Researchers. Annals of Family Medicine 2004;2(3):204–08 15209195 10.1370/afm.141 PMC1466680

[56] Hussey MA, Hughes JP. Design and analysis of stepped wedge cluster randomized trials. Contemp Clin Trials. 2007;28(2):182–91. 16829207 10.1016/j.cct.2006.05.007

[57] Husebo BS, Ballard C, Sandvik R, Nilsen OB, Aarsland D. Efficacy of treating pain to reduce behavioural disturbances in residents of nursing homes with dementia: cluster randomised clinical trial. BMJ. 2011;343:d4065 10.1136/bmj.d4065 21765198 PMC3137923

[58] French DD, Campbell R, Spehar A, Cunningham F, Foulis P. (2005) Outpatient Medications and Hip Fractures in the US: A National Veterans Study. Drugs and Aging. Vol. 22(10) pp. 877–885. 10.2165/00002512-200522100-00006 16245960

[59] van Doorn C, Gruber-Baldini A, Zimmermann S, Hebel JR, Port CL, Baumgarten M, et al Dementia as a risk factor for falls and fall injuries among nursing home residents. *Journal of the American Geriatrics Society* 2003; 51: 1213–1218 12919232 10.1046/j.1532-5415.2003.51404.x

[60] Sørensen HJ, Jensen SO, Nielsen J. Schizophrenia, antipsychotics and risk of hip fracture: a population-based analysis. Eur Neuropsychopharmacol. 2013;23(8):872–8. 10.1016/j.euroneuro.2013.04.002 Epub 2013 May 1. . 23642346

[61] Rosenberg PB, Mielke MM, Han D, Leoutsakos JS, Lyketsos CG, Rabins PV, et al The association of psychotropic medication use with the cognitive, functional, and neuropsychiatric trajectory of Alzheimer's disease. International journal of geriatric psychiatry, 2012;27(12), 1248–1257. 10.1002/gps.3769 22374884 PMC3448859

[62] Barry HE, Parsons C, Passmore AP, Hughes CM. Pain in care home residents with dementia: an exploration of frequency, prescribing and relatives' perspectives. Int J Geriatr Psychiatry 2015;30(1):55–63. 10.1002/gps.4111 24733695

[63] Bucks RS, Ashworth DL, Wilcock GK, Siegfried K. Assessment of activities of daily living in dementia: development of the Bristol Activities of Daily Living Scale. Age and Ageing 1996;25: 113–120. 8670538 10.1093/ageing/25.2.113

[64] Allen NH, Gordon S, Hope T, Burns A. Manchester and Oxford Universities Scale for the Psychopathological Assessment of Dementia (MOUSEPAD). The British Journal of Psychiatry, 1996;169(3), 293–307. 8879715 10.1192/bjp.169.3.293

[65] Act, Mental Capacity. The Stationery Office. 2005.

[66] Weijer C, Grimshaw JM, Eccles MP, McRae AD, White A, Brehaut JC, et al Ottawa Ethics of Cluster Randomized Trials Consensus Group. The Ottawa Statement on the Ethical Design and Conduct of Cluster Randomized Trials. PLoS Med. 2012;9(11):e1001346. 23185138 10.1371/journal.pmed.1001346 PMC3502500

[67] Diazordaz K, Slowther AM, Potter R, Eldridge S. Consent processes in cluster-randomised trials in residential facilities for older adults: a systematic review of reporting practices and proposed guidelines. BMJ Open. 2013;3(7). pii: e003057 10.1136/bmjopen-2013-003057 23836761 PMC3710983

[68] Council of International Organisations of Medical Sciences (CIOMS). International Ethical Guidelines for Biomedical Research Involving Human Subjects. Published in Geneva, Switzerland. 2002. Available: http://www.cioms.ch/publications/layout_guide2002.pdf. Accessed 4 June 2015.

[69] Curtis L PSSRU (Personal Social Services Research Unit) Unit Costs 2013. Available: http://www.pssru.ac.uk/project-pages/unit-costs/2013/. Accessed 4 June 2015.

[70] NHS Reference Costs 2012/2013. Available: https://www.gov.uk/government/publications/nhs-reference-costs-2012-to-2013 Accessed 5 June 2015.

[71] Bakken MS, Engeland A, Engesæter LB, Ranhoff AH, Hunskaar S, Ruths S. Risk of hip fracture among older people using anxiolytic and hypnotic drugs: a nationwide prospective cohort study. Eur J Clin Pharmacol. 2014 7;70(7):873–80. 10.1007/s00228-014-1684-z 24810612 PMC4053597

[72] European Commission. Objective 1: Supporting development in less prosperous regions. 2008. Available: http://ec.europa.eu/regional_policy/archive/objective1/index_en.htm Accessed 4 June 2015.

[73] Barber ND, Alldred DP, Raynor DK, Dickinson R, Garfield S, Jesson B, et al Care homes' use of medicines study: prevalence, causes and potential harm of medication errors in care homes for older people. Qual Saf Health Care. 2009;18(5):341–6. 10.1136/qshc.2009.034231 19812095 PMC2762085

[74] Jordan S, Coleman M, Hardy B, Hughes D. Assessing Educational Effectiveness: The impact of a specialist course on the delivery of care. Journal of Advanced Nursing 1999;30:4:796–807. 10520091 10.1046/j.1365-2648.1999.01176.x

[75] Jordan S, Watkins A, Storey M, Allen SJ, Brooks CJ, Garauova I, et al Volunteer Bias in Recruitment, Retention, and Blood Sample Donation in a Randomised Controlled Trial Involving Mothers and Their Children at Six Months and Two Years: A Longitudinal Analysis. PLoS ONE. 2013;8(7): e67912 23874465 10.1371/journal.pone.0067912 PMC3706448

[76] Puffer S, Torgerson D, Watson J. Evidence for risk of bias in cluster randomised trials: review of recent trials published in three general medical journals. BMJ. 2003;327(7418):785–9. 14525877 10.1136/bmj.327.7418.785 PMC214092

[77] McIntosh J, Stellenberg EL. Effect of a staffing strategy based on voluntary increase in working hours on quality of patient care in a hospital in KwaZulu-Natal Curations 2009;3:(2): 1 1–2 0

[78] Hwang YJ, Dixon SN, Reiss JP, Wald R, Parikh CR, Gandhi S, et al Atypical antipsychotic drugs and the risk for acute kidney injury and other adverse outcomes in older adults: a population-based cohort study. Ann Intern Med. 2014;161(4):242–8. 10.7326/M13-2796 25133360

[79] Roethlisberger FS, Dickson WJ. Management and the Worker. Harvard University Press, Cambridge, Massachusetts 1939.

[80] Sackett DL. Bias in analytic research. Journal of Chronic Diseases, 1979;32(1), 51–63. 447779 10.1016/0021-9681(79)90012-2

[81] Sackett D, Haynes RB, Guyatt G, Tugwell P. Clinical Epidemiology: A Basic Science For Clinical Medicine, 2nd ed Little, Brown, Boston, MA 1991.

[82] Boutron I, Moher D, Altman DG, Schulz KF, Ravaud P, CONSORT Group. Extending the CONSORT Statement to Randomized Trials of Nonpharmacological Treatment: Explanation and Elaboration. Annals of Internal Medicine. 2008;148: 295–309. 18283207 10.7326/0003-4819-148-4-200802190-00008

[83] Zwarenstein M, Treweek S, Gagnier JJ, Altman DG, Tunis S, Haynes B, et al Improving the reporting of pragmatic trials: an extension of the CONSORT statement. BMJ 2008;337: a2390 pp. 1223–1226. 10.1136/bmj.a2390 19001484 PMC3266844

[84] Hróbjartsson A, Thomsen AS, Emanuelsson F, Tendal B, Hilden J, Boutron I, et al Observer bias in randomised clinical trials with binary outcomes: systematic review of trials with both blinded and non-blinded outcome assessors. BMJ 2012;344: e1119 10.1136/bmj.e1119 22371859

[85] Rosenthal R, Jacobson L. Teachers' expectancies: Determinants of pupils' IQ gains. Psychological Reports, 1963;19, 115–118 10.2466/pr0.1966.19.1.115 5942071

[86] Higgins JPT, Altman DG, Sterne JAC. (editors). Assessing risk of bias in included studies Chapter 8 In: Higgins J.P.T., Green S. (editors). Cochrane Handbook for Systematic Reviews of Interventions Version 5.1.0 (updated March 2011). The Cochrane Collaboration, 2011 Available: www.cochrane-handbook.org. Accessed 4 June 2015.

[87] Schulz K, Grimes DA. The Lancet handbook of essential concepts in clinical research. Edinburgh: Elsevier 2006.

[88] Keogh-Brown MR, Bachmann MO, Shepstone L, Hewitt C, Howe A, Ramsay CR, et al Contamination in trials of education interventions. Health technology Assessment 2007;11(43): iii, ix–107. 17935683 10.3310/hta11430

[89] Christensen M, Lundh A. Medication review in hospitalised patients to reduce morbidity and mortality. Cochrane Database Syst Rev. 2013;28;2:CD008986. 10.1002/14651858.CD008986.pub2 23450593

[90] Pacini M, Smith RD, Wilson EC, Holland R. Home-based medication review in older people: is it cost effective? Pharmacoeconomics. 2007;25(2):171–80. 17249858 10.2165/00019053-200725020-00008

[91] Bosboom PR, Alfonso H, Almeida OP, Beer C. Use of Potentially Harmful Medications and Health-Related Quality of Life among People with Dementia Living in Residential Aged Care Facilities. Dement Geriatr Cogn Dis Extra. 2012;2(1):361–71. 10.1159/000342172 23277778 PMC3522451

[92] Zermansky AG, Alldred DP, Petty DR, Raynor DK, Freemantle N, Eastaugh J, et al Clinical medication review by a pharmacist of elderly people living in care homes—randomised controlled trial. Age and ageing, 2006;35(6), 586–591. 16905764 10.1093/ageing/afl075

[93] Patterson SM, Hughes C, Kerse N, Cardwell CR, Bradley MC. Interventions to improve the appropriate use of polypharmacy for older people. Cochrane Database Syst Rev, 2012;5(5). 10.1002/14651858.CD008165.pub2 22592727

[94] Viswanathan M, Kahwati LC, Golin CE, Blalock SJ, Coker-Schwimmer E, Posey R, et al Medication Therapy Management Interventions in Outpatient Settings: A Systematic Review and Meta-analysis. JAMA Intern Med. 2014; 10.1001/jamainternmed.2014.5841 [Epub ahead of print] 25401788

[95] Watson-Wolfe K, Galik E, Klinedinst J, Brandt N. Application of the Antipsychotic Use in Dementia Assessment audit tool to facilitate appropriate antipsychotic use in long term care residents with dementia. Geriatric Nursing, 2014;35(1), 71–76. 10.1016/j.gerinurse.2013.09.002 24139205

[96] Nkansah N, Mostovetsky O, Yu C, Chheng T, Beney J, Bond CM, et al Effect of outpatient pharmacists' non-dispensing roles on patient outcomes and prescribing patterns. Cochrane Database Syst Rev. 2010;(7):CD000336 10.1002/14651858.CD000336.pub2 20614422 PMC7087444

[97] Topinková E, Baeyens JP, Michel JP, Lanq PO. Evidence-based strategies for the optimization of pharmacotherapy in older people. Drugs Aging. 2012;29(6):477–94. 10.2165/11632400-000000000-00000 22642782

[98] Lapane KL, Hughes CM, Daiello LA, Cameron KA, Feinberg J. Effect of a pharmacist-led multicomponent intervention focusing on the medication monitoring phase to prevent potential adverse drug events in nursing homes. J Am Geriatr Soc. 2011;59(7):1238–45. 10.1111/j.1532-5415.2011.03418.x 21649623 PMC3157676

[99] Arditi C, Rège-Walther M, Wyatt JC, Durieux P, Burnand B. Computer-generated reminders delivered on paper to healthcare professionals; effects on professional practice and health care outcomes. Cochrane Database of Systematic Reviews 2012; Issue 12 Art. No.: CD001175 10.1002/14651858.CD001175.pub3 23235578

[100] Vigen CLP, Mack WJ, Keefe RSE, Sano M, Sultzer D, Stroup S, et al Cognitive effects of atypical antipsychotic medications in patients with Alzheimer's Disease: Outcomes From CATIE-AD. The American Journal of Psychiatry 2011;168(8)pp.831–839. 10.1176/appi.ajp.2011.08121844 21572163 PMC3310182

[101] Seitz DP, Gill SS, Herrmann N, Brisbin S, Rapoport MJ, Rines J, et al Pharmacological treatments for neuropsychiatric symptoms of dementia in long-term care: a systematic review. International Psychogeriatrics, 2013;25(02), 185–203. 23083438 10.1017/S1041610212001627 PMC3544545

[102] American Geriatrics Society Beers Criteria Update Expert Panel. American Geriatrics Society updated Beers Criteria for potentially inappropriate medication use in older adults. J Am Geriatr Soc. 2012;60:616–31 10.1111/j.1532-5415.2012.03923.x 22376048 PMC3571677

[103] Murray-Thomas T, Jones ME, Patel D, Brunner E, Shatapathy CC, Motsko S, et al Risk of mortality (including sudden cardiac death) and major cardiovascular events in atypical and typical antipsychotic users: a study with the general practice research database. Cardiovasc Psychiatry Neurol. 2013;2013:247486 10.1155/2013/247486 24455199 PMC3888674

[104] Corbett A, Burns A, Ballard C. Don’t use antipsychotics routinely to treat agitation and aggression in people with dementia. BMJ 2014;349; g6420 10.1136/bmj.g6420 25368388

[105] Bradley MC, Motterlini N, Padmanabhan S, Cahir C, Williams T, Fahey T, et al Potentially inappropriate prescribing among older people in the United Kingdom. BMC Geriatr. 2014;14:72 10.1186/1471-2318-14-72 24919523 PMC4091750

[106] Hall SA, Maserejian NN, Link CL, Steers WD, McKinlay JB. Are commonly used psychoactive medications associated with lower urinary tract symptoms? Eur J Clin Pharmacol. 2012;68(5):783–91. 10.1007/s00228-011-1170-9 22138718 PMC3538827

[107] Maughan RJ. Hydration, morbidity, and mortality in vulnerable populations. Nutr Rev. 2012;70 Suppl 2:S152–5. 10.1111/j.1753-4887.2012.00531.x 23121352

[108] Manz F, Wentz A. The importance of good hydration for the prevention of chronic diseases. Nutrition reviews, 2005;63(s1), S2–S5. 16028566 10.1111/j.1753-4887.2005.tb00150.x

[109] Aronson JK. (ed.) Meyler's Side Effects of Drugs: The International Encyclopaedia of Adverse Drug Reactions and Interactions. Amsterdam, Elsevier 2006.

[110] Golder S, Loke Y. Search strategies to identify information on adverse effects: a systematic review. Journal of the Medical Library Association 2009;97(2): 84–92. 10.3163/1536-5050.97.2.004 19404498 PMC2670220

[111] Tsang R, Colley L, Lynd LD. Inadequate statistical power to detect clinically significant differences in adverse event rates in randomized controlled trials. Journal of Clinical Epidemiology 2009;62: 609–16. 10.1016/j.jclinepi.2008.08.005 19013761

[112] Zorzela L, Golder S, Liu Y, Pilkington K, Hartling L, Joffe A, et al Quality of reporting in systematic reviews of adverse events: systematic review. BMJ. 2014;348:f7668 10.1136/bmj.f7668 24401468 PMC3898583

[113] Pieper MJ, van Dalen-Kok AH, Francke AL, van der Steen JT, Scherder EJ, Husebø BS, et al Interventions targeting pain or behaviour in dementia: a systematic review. Ageing Res Rev. 2013;12(4):1042–55. 10.1016/j.arr.2013.05.002 23727161

[114] Galvin R, Moriarty F, Cousins G, Cahir C, Motterlini N, Bradley M, et al Prevalence of potentially inappropriate prescribing and prescribing omissions in older Irish adults: findings from The Irish LongituDinal Study on Ageing study (TILDA). Eur J Clin Pharmacol. 2014 5;70(5):599–606. 10.1007/s00228-014-1651-8 Epub 2014 Feb 4. 24493365 PMC3978378

[115] Aitken M, Valkova S. Avoidable costs in US Healthcare. IMS Institute for Healthcare Informatics, Parsippany, NJ, USA 2013 Available: https://docs.google.com/gview?url=http://www.imshealth.com/deployedfiles/imshealth/Global/Content/Corporate/IMS%2520Institute/RUOM-2013/IHII_Responsible_Use_Medicines_2013.pdf&chrome=true. Accessed 4 June 2015.

[116] Bradley MC, Fahey T, Cahir C, Bennett K, O’Reilly D, Parsons C, et al Potentially inappropriate prescribing and cost outcomes for older people: a cross-sectional study using the Northern Ireland Enhanced Prescribing Database. European Journal of Clinical Pharmacology, 2012;68(10), 1425–1433. 10.1007/s00228-012-1249-y 22447297

[117] Patterson SM, Hughes CM, Cardwell C, Lapane KL, Murray AM, Crealey GE. A cluster randomized controlled trial of an adapted U.S. model of pharmaceutical care for nursing home residents in Northern Ireland (Fleetwood Northern Ireland study): a cost-effectiveness analysis. J Am Geriatr Soc. 2011;59(4):586–93. 10.1111/j.1532-5415.2011.03354.x Epub 2011 Mar 31. . 21453379

[118] Review of Health and Social Care Burdens. Lifting the Burdens Task Force. Local Government Association, London 2008.

[119] Ginsburg LR, Chuang Y, Berta WB, Norton PG, Ng P, Tregunno D, et al The relationship between organizational leadership for safety and learning from patient safety events. Health Service Research 2010;45(3),607–632. 10.1111/j.1475-6773.2010.01102.x PMC2875751 20337737

[120] National Co-ordinating Council for Medication Error Reporting and Prevention. The First Ten Years “Defining the Problem and Developing Solutions”. United States Pharmacopia, Rockville, Maryland. 2005. Available: http://www.nccmerp.org/pdf/reportFinal2005-11-29.pdf. Accessed 8 May 2013.

[121] Wightman R, Fielding J, Green S. Audit of antipsychotic prescribing in dementia: Cambridgeshire results and lessons learnt. Psychiatria Danubina 2011;23: S126–129. 21894119

[122] Hill KD, Wee R. Psychotropic drug-induced falls in older people: a review of interventions aimed at reducing the problem. Drugs Aging. 2012;29(1):15–30. 10.2165/11598420-000000000-00000 . 22191720

[123] Cleary A, Walsh F, Connolly H, Hays V, Oluwole B, Macken E, et al Monitoring and documentation of side effects from depot antipsychotic medication: an interdisciplinary audit of practice in a regional mental health service. J Psychiatr Ment Health Nurs. 2011;14 10.1111/j.1365-2850.2011.01807.x 22070791

[124] Hoffmann TC, Glasziou PP, Boutron I, Milne R, Perera R, Moher D, et al Better reporting of interventions: template for intervention description and replication (TIDieR) checklist and guide. BMJ. 2014;348:g1687 10.1136/bmj.g1687 24609605

[125] Riker GI, Setter SM. Polypharmacy in older adults at home: what it is and what to do about it-implications for home healthcare and hospice, part 2. Home Healthcare Nurse. 2013;31(2):65–77; quiz 78–9. 10.1097/NHH.0b013e31827f43b2 23385171

[126] Audit Commission. A Spoonful of Sugar: Medicines Management in NHS Hospitals. London, UK: The Stationery Office 2001.

[127] Hazell L, Shakir SA. Under-reporting of adverse drug reactions: a systematic review. Drug Safety 2006;29(5):385–96 16689555 10.2165/00002018-200629050-00003

[128] Heeley E, Riley J, Layton D, Wilton LV, Shakir SA. Prescription-event monitoring and reporting of adverse drug reactions. Lancet. 2001;358(9296):1872–3. 11741629 10.1016/S0140-6736(01)06898-2

[129] Onder G, van der Cammen TJ, Petrovic M, Somers A, Rajkumar C. Strategies to reduce the risk of iatrogenic illness in complex older adults. Age Ageing 2013;42:284–91. 10.1093/ageing/aft038 23537588

[130] McKibbon KA, Lokker C, Handler SM, Dolovich LR, Holbrook AM, O’Reily D, et al The effectiveness of integrated health information technologies across the phases of medication management: a systematic review of randomized controlled trials. Journal of the American Medical Informatics Association 2012;19: 22–30. 10.1136/amiajnl-2011-000304 21852412 PMC3240758

