# Supplementary material for: Nurse-Led Medicines' Monitoring for Patients with Dementia in Care Homes: A Pragmatic Cohort Stepped Wedge Cluster Randomised Trial
Source: PLoS One. 2015 Oct 13;10(10):e0140203. doi: 10.1371/journal.pone.0140203 (PMC4603896; doi:10.1371/journal.pone.0140203)
Supplement: S1 Appendix — (DOCX) [file pone.0140203.s001.docx]

**Appendix S1. West Wales Adverse Drug Reaction profile for medicines in mental health v. IX**

## Patient ID …………… Date _ _/_ _/_ _ Completed ___________________Profession: ________

**Authorised by** _____________________**Profession:** _________________

Circle to identify presence or absence of problems. Provide information if the problem is worsening. Care plans to be formulated / referenced on page 5. Guidelines to be stored in care notes for reference. Supporting scales are appended.

| **Section One. Vital signs. Circle as needed.** | | | | | **Problem** | | | | | | | **Actions** |
| --- | --- | --- | --- | --- | --- | --- | --- | --- | --- | --- | --- | --- |
| Heart rate | bpm | | | | no / yes | | | | | | |  |
| Irregular rhythm | | | | | no / yes | | | | | | |  |
| BP lying/ sitting | / mmHg | | | | no / yes | | | | | | |  |
| BP standing | / mHg | | | | no / yes | | | | | | |  |
| Weight / BMI | Kg kg/m^2^ | | | | no / yes | | | | | | |  |
| Change since last recording | | | | | loss / gain | | | | | | |  |
| Girth | Cm | | | | no / yes | | | | | | |  |
| Change since last recording | | | | | decrease/increase | | | | | | |  |
| Temperature (tympanic / oral / axilla / rectum) | | ^o^C | | | no / yes | | | | | | |  |
| Oxygen saturation | % | | | | no / yes | | | | | | |  |
| ECG | | | | | no / yes | | | | | | | date last recorded __/__/__ |
| **Section Two. Observations of problems. Circle as needed.** | | | | | | | | | **Actions** | | | |
| Hand tremor affecting drinking, OR eating, OR doing up buttons / zips | | | | no / yes / worse | | | | | If Yes, ADL score | | | |
| Tongue tremor | | | | no / yes / worse | | | | | If yes, AIMS score __ | | | |
| Feet shuffling | | | | no / yes / worse | | | | | If yes, Barnes’ score __ | | | |
| Abnormal movements | | | | no / yes / worse | | | | | If yes, AIMS / Barnes’ score __ | | | |
| Posture abnormal | | | | no / yes / worse | | | | | If yes, EPS score __ | | | |
| Gait abnormal on walking | | | | no / yes / worse | | | | |  | | | |
| Balance/ co-ordination poor, affects any ADLs | | | | no / yes / worse | | | | | If Yes, ADL score | | | |
| ANY bleeding/ bruising/ nosebleeds | | | | no / yes / worse | | | | |  | | | |
| Feeling the cold | | | | no / yes / worse | | | | |  | | | |
| Cognitive decline/ memory, concentration problem | | | | no / yes / worse | | | | | Date last recorded __/__/__ | | | |
| **Section Three. Reports of potential problems. Circle as needed.** | | | | | | | | **Actions** | | | | |
| **CNS** Any convulsions | | | no / yes / worse | | | |  | | | | | |
| Self-harm | | | no / yes / worse | | | | If yes, Barnes’ score __ date __/__/__ | | | | | |
| Physical violence to people or objects | | | no / yes / worse | | | | If yes, risk assessment date __/__/__ | | | | | |
| Aggression (including verbal) | | | no / yes / worse | | | | If yes, risk assessment date __/__/__ | | | | | |
| Irritability | | | no / yes / worse | | | |  | | | | | |
| Mood fluctuations | | | no / yes / worse | | | |  | | | | | |
| Agitation, anxiety, nervousness | | | no / yes / worse | | | |  | | | | | |
| Behaviour problems | | | no / yes / worse | | | |  | | | | | |
| Restlessness or pacing | | | no / yes / worse | | | | If yes, Barnes’ score __ | | | | | |
| Hyperactivity | | | no / yes / worse | | | |  | | | | | |
| Panic attacks | | | no / yes / worse | | | |  | | | | | |
| Confusion | | | no / yes / worse | | | |  | | | | | |
| Low energy, weakness, fatigue, apathy | | | no / yes / worse | | | |  | | | | | |
| Hallucinations / vivid dreams | | | no / yes / worse | | | |  | | | | | |
| Sleep problems/ insomnia | | | no / yes / worse | | | |  | | | | | |
| Sedation / excessive sleep >2 hours over normal | | | no / yes / worse | | | |  | | | | | |
| Dizziness | | | no / yes / worse | | | | If yes, risk of falling assessed yes/ no | | | | | |
| Falls | | | no / yes / worse | | | | If yes, where documented | | | | | |
| Headache / migraine | | | no / yes / worse | | | |  | | | | | |
| Tinnitus / hearing problems | | | no / yes / worse | | | |  | | | | | |
| Tingling / pins & needles | | | no / yes / worse | | | |  | | | | | |
| Urination problems /incontinence | | | no / yes / worse | | | |  | | | | | |
| Burning / discomfort on urination / UTI | | | no / yes / worse | | | |  | | | | | |
| *Discretionary question* Reproductive system e.g. breast discomfort, erectile dysfunction, change in libido | | | no / yes / worse | | | |  | | | | | |
| **Heart** Chest pain | | | no / yes / worse | | | |  | | | | | |
| Short of breath/ unable to finish sentences | | | no / yes / worse | | | |  | | | | | |
| **GI tract** Hypersalivation / frequent change of clothes due to drooling | | | no / yes / worse | | | | If yes, respiratory tract infections yes/no | | | | | |
| Nausea / vomiting | | | no / yes / worse | | | |  | | | | | |
| Appetite/ taste changes | | | no / yes / worse | | | |  | | | | | |
| Bowel control/ diarrhoea | | | no / yes / worse | | | | If yes, list laxatives used | | | | | |
| Constipation (Bowels not open in last 36 hours) | | | no / yes / worse | | | |  | | | | | |
| **Skin** Rash (+/- itching) | | | no / yes / worse | | | |  | | | | | |
| Swelling/ oedema | | | no / yes / worse | | | | If yes, Waterlow score date __ / __ / | | | | | |
| Sweating | | | no / yes / worse | | | | If yes, Waterlow score date __ / __ / | | | | | |
| Injection site e.g. pain | | | no / yes / worse | | | | NA | | | | | |
| **Section Four: Health promotion. Circle as needed.** | | | | | | | | | | **Actions** | | |
| **Intake** Misses any meals or leaves unfinished more than once a day? | | | | | | no/yes | | | | |  | |
| ‘Snacking’ or eating between meals? | | | | | | no/yes | | | | | Examples: | |
| Takes at least 1 pint of milk/ day? Include milk added to drinks/meals/cereals | | | | | | yes/no | | | | |  | |
| Vitamin D intake adequate (sunlight exposure, eating oily fish) | | | | | | yes/no | | | | |  | |
| Eating fruit or vegetables every day? | | | | | | yes/no | | | | |  | |
| Drinking at least 6-8 cups/ day? Includes water, tea, etc | | | | | | yes/no | | | | |  | |
| Are drinks sugar free? | | | | | | yes/no | | | | | If no, weight normal: yes/no | |
| Swallowing difficulties | | | | | | no/yes | | | | | If yes, date assessed __/__/__ | |
| Indigestion or heartburn | | | | | | no/yes | | | | | If yes, name medicines used | |
| **Dentists & Oral care**  Problems with teeth or dentures | | | | | | no/yes | | | | |  | |
| Dry mouth | | | | | | no/yes | | | | | If yes, in care plan: yes/no | |
| Halitosis (bad breath) | | | | | | no/yes | | | | |  | |
| Dentist visit in last 12 months | | | | | | yes/no | | | | |  | |
| **Opticians** Vision problems / dry eyes / conjunctivitis | | | | | | no/yes | | | | |  | |
| Optician visit in last 12 months | | | | | | yes/no | | | | |  | |
| **Skin** Is sunscreen of 4 stars and high factor available? | | | | | | yes/no | | | | |  | |
| Is it applied evenly? | | | | | | yes/no | | | | |  | |
| Does the client wear dark glasses in bright sunlight? | | | | | | yes/no | | | | |  | |
| Hair loss | | | | | | no/yes | | | | |  | |
| Acne / *herpes simplex* (cold sores) | | | | | | no/yes | | | | | If yes, advice sought: yes/no | |
| **Pain** Any pain? E.g. joint pain. | | | | | | no/yes | | | | | List medicines used | |
| Non-verbal pain indicators checked? | | | | | | yes/no | | | | |  | |
| **Medicines administration** Regular medication taken at the same time each day? | | | | | | yes/no | | | | |  | |
| More than 2 doses of prescribed medicines missed over any period of seven days in the last month? (including refusal) | | | | | | no/yes | | | | |  | |

**The ADR profile identifies potential adverse effects of antipsychotic, antidepressant and anti-epileptic medicines. Prescribers should be informed if adverse effects are identified. The** profile should be discussed at the next medication review.

**Medicines currently prescribed** Please include inhalers, creams, supplements and vitamins. Attach copy of drug chart if preferred. Information can be gathered by researchers or added by care staff, at the discretion of care staff.

| Drug | Dose | Taken at (Times of day) | Date started or on arrival (OA) | Prescriber | Date last modified |
| --- | --- | --- | --- | --- | --- |
|  |  |  |  | GP/consultant |  |
|  |  |  |  | GP/consultant |  |
|  |  |  |  | GP/consultant |  |
|  |  |  |  | GP/consultant |  |
|  |  |  |  | GP/consultant |  |
|  |  |  |  | GP/consultant |  |
|  |  |  |  | GP/consultant |  |
|  |  |  |  | GP/consultant |  |
|  |  |  |  | GP/consultant |  |
|  |  |  |  | GP/consultant |  |
|  |  |  |  | GP/consultant |  |

| **Section five. Tests performed in the last year. Report abnormalities and repeat test. Monitor borderline results. This section should be completed from the patient’s notes** | | | | |
| --- | --- | --- | --- | --- |
| **Tests in the last year** | | Normal | Action | |
| *Glucose | | yes / no |  | |
| CPK | | yes / no |  | |
| *FBC/ Hb | | yes / no |  | |
| *Platelets | | yes / no |  | |
| *White cells | | yes / no |  | |
| Serum and RBC Folate | | yes / no |  | |
| Serum B12 | | yes / no |  | |
| *Lipid profile | | yes / no |  | |
| *Sodium | | yes / no |  | |
| *Potassium | | yes / no |  | |
| Calcium (corrected) | | yes / no |  | |
| *Urea | | yes / no |  | |
| *Creatinine | | yes / no |  | |
| *LFTs | | yes / no |  | |
| TFTs | | yes / no |  | |
| *Prolactin | | yes / no |  | |
| Plasma protein | | yes / no |  | |
| Drug concentrations monitored (please name drug and check therapeutic range) | | yes / no |  | |
| Bone density assessments in last 5 years  (anti-epileptic and anti-psychotic drugs) | | yes / no |  | |
| *Recommended or advised in BNF (2012, 64)  Baseline plus regular intervals, depending on drug | |  |  | |
| Section six. Action plan. Please record intended actions and formulate a care plan based on problems identified. Refer to existing care plans as needed. Please see guidelines for further information | | | |  |
| Problem | **Care plan** | | |  |

## Other problems

## Continue on separate sheet if needed and append.

*© Sue Jordan 1999 / 2002/2007/ 2008/ 2014*
