# Supplementary material for: Nurse-Led Medicines' Monitoring for Patients with Dementia in Care Homes: A Pragmatic Cohort Stepped Wedge Cluster Randomised Trial
Source: PLoS One. 2015 Oct 13;10(10):e0140203. doi: 10.1371/journal.pone.0140203 (PMC4603896; doi:10.1371/journal.pone.0140203)
Supplement: S2 Appendix — (DOCX) [file pone.0140203.s002.docx]

**S2 Appendix. The West Wales Adverse Drug Reaction (WWADR) Profile for Mental Health Medicines: template for intervention description and replication (TIDieR) checklist (Hoffmann et al 2014)**

| Item No | Item | WWADR  Profile |
| --- | --- | --- |
| **Brief name** | |  |
| 1 | Provide the name or a phrase that describes the intervention | WWADR  Profile |
| **Why** | |  |
| 2 | Describe any rationale, theory, or goal of the elements essential to the intervention | Patient safety, recognition of putative ADRs |
| **What** | |  |
| 3 | Materials: Describe any physical or informational materials used in the intervention, including those provided to participants or used in intervention delivery or in training of intervention providers. Provide information on where the materials can be accessed (such as online appendix, URL) | See Jordan et al 2004, 2014 and S3 for this study |
| 4 | Procedures: Describe each of the procedures, activities, and/or processes used in the intervention, including any enabling or support activities | Nurses complete the Profile with patients, in stages if necessary |
| **Who provided** | |  |
| 5 | For each category of intervention provider (such as psychologist, nursing assistant), describe their expertise, background, and any specific training given | Guidelines provided |
| **How** | |  |
| 6 | Describe the modes of delivery (such as face to face or by some other mechanism, such as internet or telephone) of the intervention and whether it was provided individually or in a group | Face to face  Individually |
| **Where** | |  |
| 7 | Describe the type(s) of location(s) where the intervention occurred, including any necessary infrastructure or relevant features | Any healthcare setting |
| **When and How Much** | |  |
| 8 | Describe the number of times the intervention was delivered and over what period of time including the number of sessions, their schedule, and their duration, intensity, or dose | Every 4 weeks |
| **Tailoring** | |  |
| 9 | If the intervention was planned to be personalised, titrated or adapted, then describe what, why, when, and how | At the discretion of clinicians |
| **Modifications** | |  |
| 10* | If the intervention was modified during the course of the study, describe the changes (what, why, when, and how) | NA |
| **How well** | |  |
| 11 | Planned: If intervention adherence or fidelity was assessed, describe how and by whom, and if any strategies were used to maintain or improve fidelity, describe them | Completed Profiles were assessed by researchers during data collection |
| 12* | Actual: If intervention adherence or fidelity was assessed, describe the extent to which the intervention was delivered as planned | Some monitoring of vital signs was incomplete, table 2 |
