# Supplementary material for: Nurse-Led Medicines' Monitoring for Patients with Dementia in Care Homes: A Pragmatic Cohort Stepped Wedge Cluster Randomised Trial
Source: PLoS One. 2015 Oct 13;10(10):e0140203. doi: 10.1371/journal.pone.0140203 (PMC4603896; doi:10.1371/journal.pone.0140203)
Supplement: S1 Protocol — (PDF) [file pone.0140203.s004.pdf]

Welcome to the Integrated Research Application System

IRAS Project Filter

The integrated dataset required for your project will be created from the answers you give to the following questions. The system will generate only those questions and sections which (a) apply to your study type and (b) are required by the bodies reviewing your study. Please ensure you answer all the questions before proceeding with your applications.

Please enter a short title for this project (maximum 70 characters)
Nurse-led medication monitoring: a feasibility study

1. Is your project research?

(•) Yes ( ) No

2. Select one category from the list below:

( ) Clinical trial of an investigational medicinal product
( ) Clinical investigation or other study of a medical device
( ) Combined trial of an investigational medicinal product and an investigational medical device
( ) Other clinical trial to study a novel intervention or randomised clinical trial to compare interventions in clinical practice
( ) Basic science study involving procedures with human participants
( ) Study administering questionnaires/interviews for quantitative analysis, or using mixed quantitative/qualitative methodology
( ) Study involving qualitative methods only
( ) Study limited to working with human tissue samples (or other human biological samples) and data (specific project only)
( ) Study limited to working with data (specific project only)
( ) Research tissue bank
( ) Research database

If your work does not fit any of these categories, select the option below:

(•) Other study

2a. Will the study involve the use of any medical device without a CE Mark, or a CE marked device which has been modified or will be used outside its intended purposes?

( ) Yes (•) No

2b. Please answer the following question(s):

a) Does the study involve the use of any ionising radiation?	( ) Yes	(•) No
b) Will you be taking new human tissue samples (or other human biological samples)?	( ) Yes	(•) No
c) Will you be using existing human tissue samples (or other human biological samples)?	( ) Yes	(•) No

3. In which countries of the UK will the research sites be located? *(Tick all that apply)*

[ ] England

- [ ] Scotland
- [x] Wales
- [ ] Northern Ireland

3a. In which country of the UK will the lead NHS R&D office be located:

- ○ England
- ○ Scotland
- ◉ Wales
- ○ Northern Ireland
- ○ This study does not involve the NHS

4. Which review bodies are you applying to?

- [x] NHS/HSC Research and Development offices
- [ ] Social Care Research Ethics Committee
- [x] Research Ethics Committee
- [ ] National Information Governance Board for Health and Social Care (NIGB)
- [ ] Ministry of Justice (MoJ)
- [ ] National Offender Management Service (NOMS) (Prisons & Probation)

For NHS/HSC R&D offices, the CI must create Site-Specific Information Forms for each site, in addition to the study-wide forms, and transfer them to the PIs or local collaborators.

5. Will any research sites in this study be NHS organisations?

◉ Yes ○ No

6. Do you plan to include any participants who are children?

○ Yes ◉ No

7. Do you plan at any stage of the project to undertake intrusive research involving adults lacking capacity to consent for themselves?

◉ Yes ○ No

Answer Yes if you plan to recruit living participants aged 16 or over who lack capacity, or to retain them in the study following loss of capacity. Intrusive research means any research with the living requiring consent in law. This includes use of identifiable tissue samples or personal information, except where application is being made to the NIGB Ethics and Confidentiality Committee to set aside the common law duty of confidentiality in England and Wales. Please consult the guidance notes for further information on the legal frameworks for research involving adults lacking capacity in the UK.

8. Do you plan to include any participants who are prisoners or young offenders in the custody of HM Prison Service or who are offenders supervised by the probation service in England or Wales?

○ Yes ◉ No

9. Is the study or any part of it being undertaken as an educational project?

○ Yes ◉ No

10. Will this research be financially supported by the United States Department of Health and Human Services or any of its divisions, agencies or programs?

○ Yes ◉ No

11. Will identifiable patient data be accessed outside the care team without prior consent at any stage of the project (including identification of potential participants)?

○ Yes ◉ No

Integrated Research Application System
Application Form for Other research

National Patient Safety Agency

National Research Ethics Service

Application to NHS/HSC Research Ethics Committee

The Chief Investigator should complete this form. Guidance on the questions is available wherever you see this symbol displayed. We recommend reading the guidance first. The complete guidance and a glossary are available by selecting Help.

Please define any terms or acronyms that might not be familar to lay reviewers of the application.

Short title and version number: (maximum 70 characters - this will be inserted as header on all forms)
Nurse-led medication monitoring: a feasibility study

Please complete these details after you have booked the REC application for review.

REC Name:
South West Wales Research Ethics Committee

REC Reference Number:
13/WA/0067

Submission date:
28/02/2013

PART A: Core study information

1. ADMINISTRATIVE DETAILS

A1. Full title of the research:

Nurse-led medication monitoring for patients with dementia in care homes in South West Wales: a feasibility study for a stepped wedge trial (version 1).

A3-1. Chief Investigator:

	Title	Forename/Initials	Surname
	Dr	S.E	Jordan

Post	Reader
Qualifications	MB.BCh., PhD., PGCE (FE), FHEA
Employer	Swansea University
Work Address	College of Human and Health Sciences,
	Swansea Univeristy
	Singleton Park
Post Code	SA2 8PP
Work E-mail	s.e.jordan@swansea.ac.uk
* Personal E-mail	

Work Telephone 01792518541
* Personal Telephone/Mobile
Fax

** This information is optional. It will not be placed in the public domain or disclosed to any other third party without prior consent.*
A copy of a current CV (maximum 2 pages of A4) for the Chief Investigator must be submitted with the application.

A4. Who is the contact on behalf of the sponsor for all correspondence relating to applications for this project?
This contact will receive copies of all correspondence from REC and R&D reviewers that is sent to the CI.

	Title	Forename/Initials	Surname
	Dr	Susan	Jordan

Address: College of Human and Health Sciences
Swansea University
Singleton Park
Post Code: SA2 8PP
E-mail: s.e.jordan@swansea.ac.uk
Telephone: 01792518541
Fax:

A5-1. Research reference numbers. *Please give any relevant references for your study:*

Applicant's/organisation's own reference number, e.g. R & D (if available):	Pending
Sponsor's/protocol number:	Pending
Protocol Version:	1
Protocol Date:	
Funder's reference number:	
Project website:	

Registry reference number(s):
The Department of Health's Research Governance Framework for Health and Social Care and the research governance frameworks for Wales, Scotland and Northern Ireland set out the requirement for registration of trials. Furthermore: Article 19 of the World Medical Association Declaration of Helsinki adopted in 2008 states that "every clinical trial must be registered on a publicly accessible database before recruitment of the first subject"; and the International Committee of Medical Journal Editors (ICMJE) will consider a clinical trial for publication only if it has been registered in an appropriate registry. Please see guidance for more information.

International Standard Randomised Controlled Trial Number (ISRCTN):
ClinicalTrials.gov Identifier (NCT number):

Additional reference number(s):

Ref.Number Description	Reference Number

A5-2. Is this application linked to a previous study or another current application?

(•) Yes ( ) No

Please give brief details and reference numbers.
This application is a follow-on project from earlier work (12/WA/0311) exploring nurse-led medication monitoring in care homes.

2. OVERVIEW OF THE RESEARCH

To provide all the information required by review bodies and research information systems, we ask a number of specific questions. This section invites you to give an overview using language comprehensible to lay reviewers and members of the public. Please read the guidance notes for advice on this section.

A6-1. Summary of the study. *Please provide a brief summary of the research (maximum 300 words) using language easily understood by lay reviewers and members of the public. Where the research is reviewed by a REC within the UK Health Departments Research Ethics Service, this summary will be published on the website of the National Research Ethics Service following the ethical review.*

National Dementia Strategies highlight the importance of improving the quality of care for people with dementia, emphasising the delivery of person-centred care and reduction in inappropriate use of medications.

Most adverse reactions to long-term medicines could be ameliorated by regularly monitoring patients for known adverse effects. We propose to introduce this using the West Wales Adverse Drug Reaction (ADR) Profile for Mental Health medicines*. This comprises a checklist of questions, and nursing observations (including weight, blood pressure), based on the known adverse effects of mental health medicines. The first section can be passed to the prescriber, with problems highlighted, while the health promotion section is passed to nurses or carers with suggestions for actions. Guidelines are supplied.
Our research question is: “Does introduction of medication monitoring improve clinical outcomes?” We are interested in the effects of medication monitoring on clinical outcomes, patient functioning, changes in documentation and use of medicines.
Five care homes in the public and private sector will use the West Wales (ADR) Profile with 10 patients each at randmoised and stepped intervals. Care home staff will complete the ADR profile during usual care*. This will involve observing or questioning patients and seeking information from medical records.
Participants will be asked to allow researchers to access their case notes for information relevant to the project. Most patients will not be able to give informed consent, and we shall seek assent from their guardians. Verbal consent/assent will be obtained by qualified care home staff, in the presence of a witness, who it not part of the research team.
Data will be extracted from the patients’ notes for evidence of medication monitoring and actions taken. Staff will be asked to provide some feedback at the end of the project during short interviews, whcih will be audio-recorded and transcribed.
*The ADR Profile. already available in the public domain, will be used in the care home as an education package (Jordan 2008, Jordan et al 2004).

A6-2. Summary of main issues. *Please summarise the main ethical, legal, or management issues arising from your study and say how you have addressed them.*

Not all studies raise significant issues. Some studies may have straightforward ethical or other issues that can be identified and managed routinely. Others may present significant issues requiring further consideration by a REC, R&D office or other review body (as appropriate to the issue). Studies that present a minimal risk to participants may raise complex organisational or legal issues. You should try to consider all the types of issues that the different reviewers may need to consider.

No physical or emotional risks entailed. No relationship to declare with participants. No interests to declare, and no commercial involvement.

Participants
We are aware that many of the participants involved in this project are likely to lack the capacity to consent to the researcher accessing their case notes. In this instance the qualified staff, who have evidence of GCP training or are familiar with the Mental Capacity Act 2005, will select and approach potential participants. Consent will not be coerced and if necessary the participant will have up to 14 days to decide if they would like to be involved. If capacity is deemed to be lacking by the trained staff, then the participants' guardian will be approached by the qualified staff for the purposes of giving verbal assent for their relative to be involved in the study. The participants' general practitioner will also be made aware of the study in writing.

Our study is client-centred and all those involved, whether they choose to withdraw or not, will receive the utmost care. Benefits to the individual are minimal in this research, however the project will provide dedicated time for the patients, relatives or carers to discuss their experience of medication use and to highlight any concerns they may have. This will be done with the reassurance that trained staff are available when needed to provide support. Any problems identified will be noted and actioned by the nursing staff. The nurse will refer any problems as necessary to the prescriber and other members of the healthcare team.

Few risks are perceived to participants as they will continue to receive the usual and standard care of the care home. However, participants may have extra contact with the nursing staff when completing the West Wales ADR Profile. All nurse-patient consultations will take place at a time and pace to suit the participant, and not interfere with routine care.

If the participant becomes anxious as a result of the administration of the West Wales ADR Profile, the nursing staff will be able to discontinue / pause completion of the profile. When the participant feels able, the nurse and participant will be able to revisit the profile. The trained staff in the care home are prepared to counsel and support participants throughout the project.

Participants will be supported to discontinue involvement in the project should they express the wish to do so. Usual care will remain upon completion of the project. All data will be kept secured and confidentiality maintained.

Staff
Extra time spent completing the West Wales ADR Profile may be an important consideration. However, the nurses are able to use the profile at times that suit their routine and over a number of consultations with participants, if appropriate. Previous work has indicated that the profile takes 20-25 minutes to implement in this client group. For the purposes of the research, staff will also be asked to complete standard instruments on 6 occasions: Bristol Activities of Daily Living Scale plus an assessment of behaviour symptoms.

Staff who are interested in taking part in the interviews, will be asked to spare an hour for the interviews or group interviews at their place of work, during their own time. The time needed to collect clinician feedback in interviews or group interviews will occur at a suitable time for the staff and the researcher will travel to the participants place of work.

Researchers in the field
Potential risk to the researchers is perceived to be minimal. There is likely to be little lone researcher-participant interaction. If the participant wishes to speak with the researcher, the nurse will also be present. As the main researcher in the field is a qualified nurse it is recognised that this may have the potential to raise a conflict of interest between the roles of researcher and nurse. The researcher will not be directly responsible for delivering care but any identified problems or adverse events will be reported to the care home nursing staff and/or manager. The researcher will keep a log of any adverse events. The researcher will practice under the NMC code of conduct and conform with GCP.

3. PURPOSE AND DESIGN OF THE RESEARCH

A7. Select the appropriate methodology description for this research. *Please tick all that apply*:

- [x] Case series/ case note review
- [ ] Case control
- [ ] Cohort observation
- [ ] Controlled trial without randomisation
- [ ] Cross-sectional study
- [ ] Database analysis
- [ ] Epidemiology
- [x] Feasibility/ pilot study
- [ ] Laboratory study
- [ ] Metanalysis
- [ ] Qualitative research
- [x] Questionnaire, interview or observation study
- [x] Randomised controlled trial
- [ ] Other (please specify)

A10. What is the principal research question/objective? *Please put this in language comprehensible to a lay person.*

Overall, our research examines if structured nurse-led medication monitoring improves clinical outcomes for people with dementia prescribed antipsychotic, antidepressant or antiepileptic drugs in care homes. This work builds on the success of previous projects (Jordan 2002, Jordan et al. 2002, 2004, Gabe PhD thesis 2012). The primary research

question of this stepped wedge study is the feasibility of introducing nurse-led medication monitoring to a number of care homes at stepped intervals, in preparation for a larger trial. Using case note review, the study will seek to idenitfy any new problems found since using the ADR profile, any immedicate actions taken, and whether any problems identified are followed through, changes in patients' functioning as indicated by the patients' records and measured by standard instruments: Bristol Activities of Daily Living Scale plus an assessment of behaviour symptoms (Mousepad).

A11. What are the secondary research questions/objectives if applicable? *Please put this in language comprehensible to a lay person.*

The feasibility of a stepped wedge trial in nurse-led medication monitoring, including rates of recruitment, attrition, cross-over, profile completion, compliance will also be explored, along with the views of clinicians involved. This will help us to assess the best way to measure any impact of nurse-led medication monitoring using a structured adverse drug reaction profile in terms of patient problems found, actions taken, clinical gain, patient functioning, and use of medications.

A12. What is the scientific justification for the research? *Please put this in language comprehensible to a lay person.*

Patient safety is a priority for healthcare organisations, but there are underlying weaknesses in current practice, particularly medication monitoring for known adverse effects of prescribed drugs (NPSA 2007, HSC 2011, Gabe et al 2011, Jordan 2011). 4-6% of hospital admissions are due to adverse drug reactions* (ADRs), most of which are preventable (reviewed Jordan 2007, 2011). Failure to monitor for common problems, rather than poor prescribing, is responsible for the majority of ADRs (Forster et al 2005, Gurwitz et al 2005, 2006, Steinman et al 2011, Gabe et al 2011, Brenner et al 2012).
Between 25-50% of people with dementia in the UK are prescribed antipsychotic medication (Banerjee 2009), but there is international variation (Testad et al 2010). For people with dementia, some antipsychotics reduce aggression and psychosis (Ballard et al 2006, 2008, Sultzer et al 2009), particularly amongst those most severely agitated (Katz et al 2007). However, in older people, antipsychotics are associated with: increased overall mortality (Schneider et al 2005, Ballard et al 2009, Smith 2011), worsening cognitive impairment (Vigen et al 2011), hip fracture (French et al 2005, Pratt et al 2011), diabetes (Jalbert et al 2011) and stroke (Banerjee 2009). Withdrawal of medication reduces falls (Iyer et al 2008), and improves verbal fluency (Ballard et al 2008), but aggressive behaviour may return (Devanand et al 2011).
Over one third of care home residents receive antidepressants (Harris et al 2012), sometimes longer than necessary (Jureidini & Tonkin 2006). Depressive co-morbidity is common amongst those with dementia and anti-depressants have known benefits. However, their use amongst older adults is associated with serious adverse events (Coupland et al 2011): serious bleeding (Lewis et al 2008), violent behaviour (Healy et al 2006), falls (Masud et al 2012), fractures (French et al 2005, Rabenda et al 2012). In older adults, more subtle adverse effects, such as polyuria, insomnia or wandering may predominate, which will only be uncovered by structured monitoring (Jordan 2008).

Some 10-20% patients with Alzheimer’s suffer seizures. Older adults are particularly vulnerable to CNS depression and other adverse effects of AEDs (Stefan 2011).

The immediate difficulties of managing challenging behaviour or depression may be seen as more pressing than possible ADRs or the small increase in mortality associated with antipsychotics, 2.3% vs 3.5% (OR 1.54, 1.06-2.23) over 10-12 weeks (Schneider et al 2005). Although effective, non-pharmacological interventions are not always deployed (Fossey et al 2006, Alzheimer’s Society 2011). While practitioners are warned against prescribing antipsychotics to those with dementia (FDA 2008, BNF 2012), no recommendations on monitoring are offered. Further research into increased nursing vigilance and improved systems for actively monitoring patients for known adverse effects of prescribed medicines is needed (NCC 2005; Herxheimer & Sanz 2008, Jordan 2009, 2011, Thompson et al 2011, Wightman et al 2011, Steinman et al 2011, Hill & Wee 2012), and monitoring profiles are ideal (Cleary et al 2011). Previous work, observing and questioning 20 adult mental health clients indicated that orthostatic hypotension, coupled beats, hypertension, constipation, inadequate diet were previously undetected (Jordan 2002, Jordan et al 2002). We aim to explore how highlighting problems can be translated into clinical gains for older adults in long-term care, adapting our existing profiles (Jordan et al 2004, Jordan 2008, Gabe et al 2011).
* Footnote. An adverse drug reaction is defined as any untoward and unintended response in a patient or investigational subject to a medicinal product which is related to any dose administered (ICH 1996).

A13. Please summarise your design and methodology. *It should be clear exactly what will happen to the research participant, how many times and in what order. Please complete this section in language comprehensible to the lay person. Do not simply reproduce or refer to the protocol. Further guidance is available in the guidance notes.*

Five care homes will be asked to use the West Wales ADR Profile for mental health drugs (Jordan et al. 2004), modified following pre-pilot work, for implementation with 10 patients each. Each care home will start using the ADR profile in a stepped wedge fashion. The researcher will contact each care home to ask them to start using the ADR

profile. Care home staff will be asked to complete the ADR profile during their usual care and contact with patients. This may involve observing or questioning patients several times and seeking information from medical records. In addion to the ADR profile, care homes will be asked to monitor level of functioning, measured by standard instruments: Bristol Activities of Daily Living Scale plus an assessment of behaviour symptoms on 6 occasions + complete the profile on each occasion after assignment (figure attached). Health care support workers (who work with / know the patients and are therefore able, with the patients permission, to report health problems) may also be approached to provide details of signs and symptoms of potential adverse effects of the medications.

Potential participants will be asked to give verbal assent/ consent to the researchers accessing their case notes for information directly relevant to the project. We are aware that some/most patients may not be able to give informed consent and we would seek verbal assent from guardians. Consent/assent will be obtained by qualified care home staff who are able to obtain informed consent for research purposes. Evidence of Good Clinical Practice training and / or familiarity with the Mental Capacity Act (2005) will be sought. Letters introducing the project and outlining the involvement of the participant will be sent to participants' General Practitioners (attached). Verbal consent/assent will be sought in the presence of a witness/consultee, who is not part of the research team. The witness/consultee will be asked to sign witness to the consent/assent. A letter of infomarion will be posted to the participants' registered general practitioner.

Researchers will then extract data from the patients' case notes to look for evidence of medication monitoring, any actions emanating from this, and any clinical gain. No participants will be involved in the case note review.

Staff involved will then be asked to provide some comments and feedback at the end of the project during short interviews (with the potential for group interviews). Interviews will be audio recorded and transcribed. (interview schedule, including CVI attached)

A14-1. In which aspects of the research process have you actively involved, or will you involve, patients, service users, and/or their carers, or members of the public?

- [x] Design of the research
- [x] Management of the research
- [x] Undertaking the research
- [ ] Analysis of results
- [x] Dissemination of findings
- [ ] None of the above

Give details of involvement, or if none please justify the absence of involvement.
Non-NHS volunteer service user representatives are already recruited and will be involved as steering group members throughout the project.

4. RISKS AND ETHICAL ISSUES

RESEARCH PARTICIPANTS

A17-1. Please list the principal inclusion criteria (list the most important, max 5000 characters).

Care home participants
No upper age limit. No specific gender criteria.

Inclusion criteria:
Resident at the care home.
Diagnosed with dementia or dementia subtypes
Currently taking at least one antipsychotic or anti-epileptic or antidepressant medicine.
Willing and able to give informed, signed consent themselves, or if capacity is lacking, guardian willing to consent to access to patient records.

Care home staff taking consent
Inclusion criteria
Registered care home staff
Qualified nursing status

Familiar with the Mental Capacity Act (2005) or attended GCP training

A17-2. Please list the principal exclusion criteria (list the most important, max 5000 characters).

Exclusion criteria:
Not be well enough to participate, as screened by their nurses.
Those aged 18 or under.

RESEARCH PROCEDURES, RISKS AND BENEFITS

A18. Give details of all non-clinical intervention(s) or procedure(s) that will be received by participants as part of the research protocol. *These include seeking consent, interviews, non-clinical observations and use of questionnaires.*

Please complete the columns for each intervention/procedure as follows:

1. Total number of interventions/procedures to be received by each participant as part of the research protocol.
2. If this intervention/procedure would be routinely given to participants as part of their care outside the research, how many of the total would be routine?
3. Average time taken per intervention/procedure (minutes, hours or days)
4. Details of who will conduct the intervention/procedure, and where it will take place.

Intervention or procedure	1	2	3	4
Patients selected by nurse	1		5 mins	Qualified nursing staff in the private care home
Written information and verbal support given out from nurses	1		10 mins	Qualified nursing staff in the private care home. Potential participants and their guardians are able to request time with the researcher, if desired.
Consent participants / assent from guardians	1		10 mins	Qualified nursing staff in the private care home (familiar with the Mental Capacity Act 2005 and/or GCP training)
Care home contacted to start medication monitoring	1		10	Each care home will be individually contacted to mark the start of medication monitoring.
Individual interviews or group interviews with staff	1-2		60 mins	Staff recruited for interviews, will be asked to complete a content validity index and to discuss their experience and views on medication monitoring. Marie Gabe and Sherrill Snelgrove at the care home, during the staffs' own time.

A19. Give details of any clinical intervention(s) or procedure(s) to be received by participants as part of the research protocol. *These include uses of medicinal products or devices, other medical treatments or assessments, mental health interventions, imaging investigations and taking samples of human biological material. Include procedures which might be received as routine clinical care outside of the research.*

Please complete the columns for each intervention/procedure as follows:

1. Total number of interventions/procedures to be received by each participant as part of the research protocol.
2. If this intervention/procedure would be routinely given to participants as part of their care outside the research, how many of the total would be routine?
3. Average time taken per intervention/procedure (minutes, hours or days).
4. Details of who will conduct the intervention/procedure, and where it will take place.

Intervention or procedure	1	2	3	4
Nurse uses the medication monitoring ADR profile as part of usual care	5	5	20-25 mins	Staff in the care home including healthcare support workers and the patients' guardian / relatives. Nurses can delegate some aspects of the profile but will remain responsible for patient care.
Behavioural assessments	6	6	15-30	Staff in the care home including healthcare support workers will

		complete an assessment once each month.
Functioning assessments	6 6 10	Staff in the care home including healthcare support workers will complete an assessment once each month.

A20. Will you withhold an intervention or procedure, which would normally be considered a part of routine care?

○ Yes ◉ No

A21. How long do you expect each participant to be in the study in total?

The participant will only be involved during their usual care as the healthcare staff complete 1) the ADR profile once each month from start in the study (care home site one will use complete the profile fives times over the course of five months, care home two four times over four months and so forth using a stepped wedge design) 2) At each month time point, the staff will complete a behavioural and functioning assessments, including at baseline. Therefore, assessments will be made on 6 occassions in total for all care homes, regardless of position in stepped wedge trial. All assessments can be done in one or more sittings to suit the participant. The profile is likely to take 20-25 minutes, depending on the number and severity of problems found. The assessments are likely to take around 10 minutes to complete. The study will run for a number of months, however, the participant does not need to have any extra contact with the researcher.

Participants also do not need to be involved during the case note review.

A22. What are the potential risks and burdens for research participants and how will you minimise them?

For all studies, describe any potential adverse effects, pain, discomfort, distress, intrusion, inconvenience or changes to lifestyle. Only describe risks or burdens that could occur as a result of participation in the research. Say what steps would be taken to minimise risks and burdens as far as possible.

There are few risks and burdens to using the ADR profile. The questions asked are of a nature that should be asked in routine practice.

Should any participant become anxious or worried, they will be supported by trained staff in the private care home. The participants' general practitioner will also be aware of the study and will be available to review the participants' medication and health. The profile will be used at a pace to suit the individual participant. Participants can discontinue involvement should they wish to.

A23. Will interviews/ questionnaires or group discussions include topics that might be sensitive, embarrassing or upsetting, or is it possible that criminal or other disclosures requiring action could occur during the study?

◉ Yes ○ No

If Yes, please give details of procedures in place to deal with these issues:

A question will be asked on whether sexual functioning has been affected by prescribed medication. This would be asked by a same sex nurse and should be asked as a part of routine care, with or without the WWADR profile. Based on previous work, this question did not appear to evoke any added distress, anxiety or concern to participants. The question on bowel function has not previously elicited embarrassment.

A24. What is the potential for benefit to research participants?

Participants will be asked a comprehensive set of questions that will ensure that all pertinent information has been gained on their physical well-being, relating to signs and symptoms of potentil adverse effects of medicines commonly prescribed for mental health conditions. Previous evidence (Jordan 2002, Jordan et al 2002) suggests that patients' quality of life may be improved and potentially life-threatening problems may be identified and addressed expeditiiously, when this tool is operationalised. functioning and behavioural assessments will be undertaken to monitor patinets' progress.

Enquiring about potential medication-related harm when using the ADR profile will help monitor the participants health and wellbeing. Any problems found will be actioned by the healthcare team. For example, oral care advice may be given or the prescriber may be asked to review the medication regimen.

A25. What arrangements are being made for continued provision of the intervention for participants, if appropriate, once the research has finished? *May apply to any clinical intervention, including a drug, medical device, mental health intervention, complementary therapy, physiotherapy, dietary manipulation, lifestyle change, etc.*

Should the care homes involved in this study wish to continue using the WWADR profile with their patients they will be supported to do so. No charges are involved.

A26. What are the potential risks for the researchers themselves? *(if any)*

Potential risk to the researchers is perceived to be minimal. There is likely to be little lone researcher-participant interaction. If the participant wishes to speak with the researcher, the nurse will also be present. As the researcher is a qualified nurse it is recognised that this may have the potential to raise a conflict of interest between the roles of researcher and nurse. The researcher will not be directly responsible for delivering care but any identified problems or adverse events will be reported to the care home nursing staff and/or manager. The researchers will log of any adverse events. The researchers will practice under the NMC code of conduct and conform with GCP. Interviews with professionals will be held on care home premsies or other mutally convenient locations.

RECRUITMENT AND INFORMED CONSENT

In this section we ask you to describe the recruitment procedures for the study. Please give separate details for different study groups where appropriate.

A27-1. How will potential participants, records or samples be identified? Who will carry this out and what resources will be used?*For example, identification may involve a disease register, computerised search of GP records, or review of medical records. Indicate whether this will be done by the direct healthcare team or by researchers acting under arrangements with the responsible care organisation(s).*

The nursing staff responsible for delivering usual care to the patients will be involved in the identification and selection of potential participants, based on inclusion criteria. Nurses will verbally introduce the project and if patients are interested, they will be given a patient information sheet. If the potential participant is considered to lack the capacity to consent, their guardian will be contacted. Patients and guardians will have up to two weeks to decide if they would like to be involved. They may involve their carer and/or families in this process. The researcher (Marie Gabe) will also be available to speak with patients if they have any concerns or questions.

Qualified staff (familiar with the Mental Capacity Act 2005 and / or GCP training) will then speak with the potential participant and seek verbal assent/ consent. Guardians of potential participants will also be approached, where relevant. Consent/assent will be given verbally either by the participant or their guardian in the presence of a witness/consultee who is not a member of the research team. The witness will then sign the consent form if the participant/guardians aggree. A copy of the consent/assent form will be secured in the case notes and a copy retained securely for research records.

A letter will be sent to the participants' general practitioner. The letter will inform the general practitioner of the study and the involvement of their patient.

The staff involved in the interviews will be approached by Marie Gabe and/or Sherrill Snelgrove. They will be invited to participate with information sheets provided. If they would like to be involved in the interviews, a consent form will be signed and a convenient time and date set for the interview. The interview will be conducted in the participants’ own time in the care home or a mutually agreed venue.

A27-2. Will the identification of potential participants involve reviewing or screening the identifiable personal information of patients, service users or any other person?

(●) Yes ( ) No

Please give details below:
The consenting staff will consult the participants' case notes to clarify that they meet the inclusion and exclusion criteria.

A27-4. Will researchers or individuals other than the direct care team have access to identifiable personal information

of any potential participants?

○ Yes ◉ No

A28. Will any participants be recruited by publicity through posters, leaflets, adverts or websites?

○ Yes ◉ No

A29. How and by whom will potential participants first be approached?

Care home nurses will approach participants, and their guardians if relevant, to introduce the study. If the participant desires the nurse will then provide an information sheet and verbal support. Consent will only be sought by those nurses who are qualified and familiar with the Mental Capacity Act 2005 and/or GCP training.

A30-1. Will you obtain informed consent from or on behalf of research participants?

◉ Yes ○ No

If you will be obtaining consent from adult participants, please give details of who will take consent and how it will be done, with details of any steps to provide information (a written information sheet, videos, or interactive material). Arrangements for adults unable to consent for themselves should be described separately in Part B Section 6, and for children in Part B Section 7.

If you plan to seek informed consent from vulnerable groups, say how you will ensure that consent is voluntary and fully informed.

All data will be anonymised and aggregated. Our pre-pilot work (12/WA/0311) has again shown that ADR profiling does not harm patients. It was reported that only better educated families gave consent for their relatives to participate. Many residents' representatives were themselves elderly, and while broadly supportive of work to improve nursing documentation and minimise adverse drug reactions, had other concerns. We suggest that signed consent might introduce volunteer bias into a larger trial, limiting generalisation of findings, statistical power and validity of conclusions (Jordan & Morgan 2011). "Where there is no more than minimal risk and requirement of individual informed consent would make the conduct of the research impracticable (for example, where research only involved excerpting data from subjects' records), the ethical review committee may waive some of all of the elements of informed consent" (CIOMS 2002 p.34). Obtaining consent is more complicated in cluster trials (McRae et al 2011). Since the WWADR profile has been in the public domain for nurses to use as part of routine care since 2004, and all questions and observations on the WWADR profile are of a nature that should be pursued under routine care, we shall seek consent for researchers to access residents' clinical notes.

Verbal consent will be obtained in the presence of and countersigned by a literate, impartial and independent witness confirming that all the relevant information was provided to the research participant in an understandable manner. The impartial witness will be independent of the trial, for example a qualified health care professional. She/ he will read the informed consent form and any other written information supplied to the subject and his / her legally acceptable representative (used to encompass terms in common parlance such as legal guardian or next of kin), and be present throughout the entire informed consent discussion.

After the written informed consent form and any other written information to be provided to subjects, is read and explained to the subject and the subject's legally acceptable representative, and after the subject and/ or the subject's legally acceptable representative (EMC 2006 p.8) has orally consented to the subject's participation in the trial, the witness will sign and personally date the consent form. By signing the consent form, the witness will attest that the information in the consent form and any other written information was accurately explained to, and apparently understood by, the subject or the subject's legally acceptable representative, and that informed consent was freely given by the subject or the subject is legally acceptable representative.

Both the informed consent discussion and the written informed consent form and any other written information to be provided to subjects / guardians will explain:
a) That the trial involves research into nursing documentation.
b) The purpose of the trial is to improve nursing documentation.
c) The documentation will be implemented with all participants in due course.
d) There are no invasive procedures.
e) The subject has no responsibilities.
f) Implementation of nursing documentation is experimental.
g) We foresee no risks or inconveniences to the subject.

h) The reasonably expected benefits are improved nursing documentation and, possibly, steps to minimize adverse drug reactions.
i) The alternative procedure(s) is not to complete the profile.
j) We foresee no potential for non-negligent harm. However, indemnity has been obtained from Swansea University.
k) There is no payment to the subjects for participating in the trial.
l) Subjects will not incur any expenses by participating in the trial.
m) That the subject's participation in the trial is voluntary and that the subject may refuse to participate or withdraw from the trial, at any time, without penalty or loss of benefits to which the subject is otherwise entitled.
n) That the auditor(s), and regulatory authority(ies) will be granted direct access to the subject's original medical records for verification of clinical trial procedures and/or data, without violating the confidentiality of the subject, to the extent permitted by the applicable laws and regulations and that the subject or the subject's legally acceptable representative is authorizing such access.
o) That records identifying the subject will be kept confidential and, to the extent permitted by the applicable laws and/or regulations, will not be made publicly available. If the results of the trial are published, the subjectís identity will remain confidential.
q) The person(s) to contact for further information regarding the trial and the rights of trial subjects, and whom to contact in the event of trial-related injury. Dr. Sue Jordan, and, if still not satisfied, Dr. Jeanette Hewitt, College of Human and Health Sciences, Swansea University.
r) The subject's participation in the trial may be terminated if she/ he leaves the care home.
s) The expected duration of the subject's participation in the trial will be up to 6 months.
t) The approximate number of subjects involved in the trial is 50.
4.8.11 Prior to participation in the trial, the subject or the subject's legally acceptable representative will receive a copy of the signed and dated written informed consent form and any other written information provided to the subjects.
4.8.12 The subject will be informed about the trial to the extent compatible with his/ her understanding. (EMA 2006 p.19)
EMA 2006 ICH Topic E 6 (R1) Guideline for Good Clinical Practice Step 5 NOTE FOR GUIDANCE ON GOOD CLINICAL PRACTICE (CPMP/ICH/135/95), Canary Warf, London available www.emea.europa.eu/pdfs/human/ich/013595en.pdf accessed 18.2.13
1.37 Legally Acceptable Representative
An individual or juridical or other body authorized under applicable law to consent, on behalf of a prospective subject, to the subject's participation in the clinical trial. P.8
References
1. GCP See www.emea.europa.eu/pdfs/human/ich/013595en.pdf
2. http://www.nres.npsa.nhs.uk/applications/guidance/#PIS
3. http://www.mrc.ac.uk/Utilities/Documentrecord/index.htm?d=MRC0024 06
4. http://www.nres.npsa.nhs.uk/news-and-publications/news/release-of -adults-lacking-capacity-toolkit/
5. http://www.nres.npsa.nhs.uk/applications/approval-requirements/et hical-review-requirements/requirements-for-ethical-review-under-l egislation/adults-lacking-capacity-to-consent-for-themselves/

1.26 Impartial Witness
A person, who is independent of the trial, who cannot be unfairly influenced by people involved with the trial, who attends the informed consent process if the subject or the subject is legally acceptable representative cannot read, and who reads the informed consent form and any other written information supplied to the subject. P.7
1.37 Legally Acceptable Representative
An individual or juridical or other body authorized under applicable law to consent, on behalf of a prospective subject, to the subject's participation in the clinical trial.
Vulnerable subjects
4.8.9 If a subject is unable to read or if a legally acceptable representative is unable to read, an impartial witness should be present during the entire informed consent discussion. After the written informed consent form and any other written information to be provided to subjects, is read and explained to the subject or the subject is legally acceptable representative, and after the subject or the subject is legally acceptable representative has orally consented to the subject is participation in the trial and, if capable of doing so, has signed and personally dated the informed consent form, the witness should sign and personally date the consent form. By signing the consent form, the witness attests that the information in the consent form and any other written information was accurately explained to, and apparently understood by, the subject or the subject's legally acceptable representative, and that informed consent was freely given by the subject or the subject is legally acceptable representative.
4.8.10 Both the informed consent discussion and the written informed consent form and any other written information to be provided to subjects should include explanations of the following:
a) That the trial involves research.
b) The purpose of the trial.
c) The trial treatment(s) and the probability for random assignment to each treatment.
d) The trial procedures to be followed, including all invasive procedures.
e) The subject's responsibilities.
f) Those aspects of the trial that are experimental.
g) The reasonably foreseeable risks or inconveniences to the subject and, when applicable, to an embryo, fetus, or

nursing infant.
h) The reasonably expected benefits. When there is no intended clinical benefit to the subject, the subject should be made aware of this.
i) The alternative procedure(s) or course(s) of treatment that may be available to the subject, and their important potential benefits and risks.
j) The compensation and/or treatment available to the subject in the event of trial-related injury.
k) The anticipated prorated payment, if any, to the subject for participating in the trial.
l) The anticipated expenses, if any, to the subject for participating in the trial.
m) That the subject's participation in the trial is voluntary and that the subject may refuse to participate or withdraw from the trial, at any time, without penalty or loss of benefits to which the subject is otherwise entitled.
n) That the monitor(s), the auditor(s), the IRB/IEC, and the regulatory authority(ies) will be granted direct access to the subject's original medical records for verification of clinical trial procedures and/or data, without violating the confidentiality of the subject, to the extent permitted by the applicable laws and regulations and that, by signing a written informed consent form, the subject or the subject's legally acceptable representative is authorizing such access.
o) That records identifying the subject will be kept confidential and, to the extent permitted by the applicable laws and/or regulations, will not be made publicly available. If the results of the trial are published, the subject is identity will remain confidential.
p) That the subject or the subject's legally acceptable representative will be informed in a timely manner if information becomes available that may be relevant to the subject's willingness to continue participation in the trial.
q) The person(s) to contact for further information regarding the trial and the rights of trial subjects, and whom to contact in the event of trial-related injury.
r) The foreseeable circumstances and/or reasons under which the subject's participation in the trial may be terminated.
s) The expected duration of the subject's participation in the trial.
t) The approximate number of subjects involved in the trial.
4.8.11 Prior to participation in the trial, the subject or the subject's legally acceptable representative should receive a copy of the signed and dated written informed consent form and any other written information provided to the subjects. During a subject is participation in the trial, the subject or the subject is legally acceptable representative should receive a copy of the signed and dated consent form updates and a copy of any amendments to the written information provided to subjects.
4.8.12 When a clinical trial (therapeutic or non-therapeutic) includes subjects who can only be enrolled in the trial with the consent of the subject is legally acceptable representative (e.g., minors, or patients with severe dementia), the subject should be informed about the trial to the extent compatible with the subject is understanding and, if capable, the subject should sign and personally date the written informed consent.
4.8.13 Except as described in 4.8.14, a non-therapeutic trial (i.e. a trial in which there is no anticipated direct clinical benefit to the subject), should be conducted in subjects who personally give consent and who sign and date the written informed consent form.
4.8.14 Non-therapeutic trials may be conducted in subjects with consent of a legally acceptable representative provided the following conditions are fulfilled:
a) The objectives of the trial cannot be met by means of a trial in subjects who can give informed consent personally.
b) The foreseeable risks to the subjects are low.
c) The negative impact on the subject is well-being is minimized and low.
d) The trial is not prohibited by law.
e) The approval/favourable opinion of the IRB/IEC is expressly sought on the inclusion of such subjects, and the written approval/ favourable opinion covers this aspect.
Such trials, unless an exception is justified, should be conducted in patients having a disease or condition for which the investigational product is intended. Subjects in these trials should be particularly closely monitored and should be withdrawn if they appear to be unduly distressed.
p.19
EMA 2006 ICH Topic E 6 (R1) Guideline for Good Clinical Practice Step 5 NOTE FOR GUIDANCE ON GOOD CLINICAL PRACTICE (CPMP/ICH/135/95), Canary Warf, London available www.emea.europa.eu/pdfs/human/ich/013595en.pdf accessed 18.2.13

If you are not obtaining consent, please explain why not.

Please enclose a copy of the information sheet(s) and consent form(s).

A30-2. Will you record informed consent (or advice from consultees) in writing?

(•) Yes ( ) No

A31. How long will you allow potential participants to decide whether or not to take part?

Participants and their guardians will have up to two weeks to decide if they want to be involved.

A32. Will you recruit any participants who are involved in current research or have recently been involved in any research prior to recruitment?

- ◯ Yes
- ◉ No
- ◯ Not Known

A33-1. What arrangements have been made for persons who might not adequately understand verbal explanations or written information given in English, or who have special communication needs?*(e.g. translation, use of interpreters)*

Information sheets will be available in large font and the staff in the care home can read the information sheets aloud to participants. The translation unit in Swansea University will be approached to translate patient material into Welsh. Verbal support will be given as needed to accommodate for any literacy needs.

A33-2. What arrangements will you make to comply with the principles of the Welsh Language Act in the provision of information to participants in Wales?

The ADR profile has been translated and is available in Welsh and English. The translation unit in Swansea University will be approached to translate patient material into Welsh.

A34. What arrangements will you make to ensure participants receive any information that becomes available during the course of the research that may be relevant to their continued participation?

Important progress information or information relating to the conduct of the study will be firstly disseminated to the care home managers and then the care home staff. Participants will then receive this information in a format suitable for them (written or spoken).

A35. What steps would you take if a participant, who has given informed consent, loses capacity to consent during the study? *Tick one option only.*

- ◉ The participant and all identifiable data or tissue collected would be withdrawn from the study. Data or tissue which is not identifiable to the research team may be retained.
- ◯ The participant would be withdrawn from the study. Identifiable data or tissue already collected with consent would be retained and used in the study. No further data or tissue would be collected or any other research procedures carried out on or in relation to the participant.
- ◯ The participant would continue to be included in the study.
- ◯ Not applicable – informed consent will not be sought from any participants in this research.
- ◯ Not applicable – it is not practicable for the research team to monitor capacity and continued capacity will be assumed.

Further details:

CONFIDENTIALITY

In this section, personal data means any data relating to a participant who could potentially be identified. It includes pseudonymised data capable of being linked to a participant through a unique code number.

Storage and use of personal data during the study

A36. Will you be undertaking any of the following activities at any stage (including in the identification of potential participants)?*(Tick as appropriate)*

- [x] Access to medical records by those outside the direct healthcare team
- [ ] Electronic transfer by magnetic or optical media, email or computer networks
- [ ] Sharing of personal data with other organisations
- [ ] Export of personal data outside the EEA
- [ ] Use of personal addresses, postcodes, faxes, emails or telephone numbers
- [x] Publication of direct quotations from respondents
- [ ] Publication of data that might allow identification of individuals
- [x] Use of audio/visual recording devices
- [ ] Storage of personal data on any of the following:
  - [ ] Manual files including X−rays
  - [ ] NHS computers
  - [ ] Home or other personal computers
  - [ ] University computers
  - [ ] Private company computers
  - [ ] Laptop computers

Further details:
Only the care home staff interviews will be audio recorded and all participant information will be anonymised, using an assigned study number.
A copy of the consent form will be retained in the participants' case notes and a copy held in a locked unit in Swansea University (separate to all other research information). Coded study numbers will be used for each participant.
The participants' general practitioners will be contacted in writing, in order to make them aware of the study.

A38. How will you ensure the confidentiality of personal data?*Please provide a general statement of the policy and procedures for ensuring confidentiality, e.g. anonymisation or pseudonymisation of data.*

Participants will be reassured that their involvement in the study is voluntary and will be confidential. The researcher, nurses and other healthcare professionals involved in this project will adhere to the relevant codes of practice, such as the Nursing and Midwifery Council Code of Conduct (2008) to ensure patient confidentiality. For this reason the staff directly involved in implementing this project will not unnecessarily disclose any patient details.

To maintain anonymity, all data collected by the researcher will be aggregated and will be coded with study numbers. Names of service users will only be recorded on the signed consent forms. This information will be secured in a locked unit, accessible only by the researcher.

A40. Who will have access to participants' personal data during the study? *Where access is by individuals outside the direct care team, please justify and say whether consent will be sought.*

Only the researcher accessing the case notes and the clinicians directly involved in caring for the participant will have access to the participants' personal information.

Storage and use of data after the end of the study

A43. How long will personal data be stored or accessed after the study has ended?

- (•) Less than 3 months
- ( ) 3 – 6 months
- ( ) 6 – 12 months
- ( ) 12 months – 3 years

◯ Over 3 years

INCENTIVES AND PAYMENTS

A46. Will research participants receive any payments, reimbursement of expenses or any other benefits or incentives for taking part in this research?

◉ Yes ◯ No

If Yes, please give details. For monetary payments, indicate how much and on what basis this has been determined.
Individual participants will not receive any reimbursement. The care home involved will be reimbursed to the sum of £200GBP to compensate for their time and involvement in the study.

A47. Will individual researchers receive any personal payment over and above normal salary, or any other benefits or incentives, for taking part in this research?

◯ Yes ◉ No

A48. Does the Chief Investigator or any other investigator/collaborator have any direct personal involvement (e.g. financial, share holding, personal relationship etc.) in the organisations sponsoring or funding the research that may give rise to a possible conflict of interest?

◯ Yes ◉ No

NOTIFICATION OF OTHER PROFESSIONALS

A49-1. Will you inform the participants' General Practitioners (and/or any other health or care professional responsible for their care) that they are taking part in the study?

◉ Yes ◯ No

If Yes, please enclose a copy of the information sheet/letter for the GP/health professional with a version number and date.

A49-2. Will you seek permission from the research participants to inform their GP or other health/ care professional?

◉ Yes ◯ No

It should be made clear in the participant's information sheet if the GP/health professional will be informed.

PUBLICATION AND DISSEMINATION

A50. Will the research be registered on a public database?

The Department of Health's Research Governance Framework for Health and Social Care and the research governance frameworks for Wales, Scotland and Northern Ireland set out the requirement for registration of trials. Furthermore: Article 19 of the World Medical Association Declaration of Helsinki adopted in 2008 states that "every clinical trial must be registered on a publicly accessible database before recruitment of the first subject"; and the International Committee of Medical Journal Editors (ICMJE) will consider a clinical trial for publication only if it has been registered in an appropriate registry. Please see guidance for more information.

◉ Yes ◯ No

Please give details, or justify if not registering the research.
The trial will be registered with ISRCTN or clinical trial.gov. This registration is pending.

Please ensure that you have entered registry reference number(s) in question A5-1.

A51. How do you intend to report and disseminate the results of the study? *Tick as appropriate:*

- [x] Peer reviewed scientific journals
- [x] Internal report
- [x] Conference presentation
- [ ] Publication on website
- [ ] Other publication
- [ ] Submission to regulatory authorities
- [ ] Access to raw data and right to publish freely by all investigators in study or by Independent Steering Committee on behalf of all investigators
- [ ]
- [ ] Other (please specify)

A53. Will you inform participants of the results?

(•) Yes ( ) No

Please give details of how you will inform participants or justify if not doing so.
An executive summary report will be available at the care home for all those involved in the study. The care home staff will offer a copy to the residents.

5. Scientific and Statistical Review

A54. How has the scientific quality of the research been assessed? *Tick as appropriate:*

- [x] Independent external review
- [ ] Review within a company
- [ ] Review within a multi−centre research group
- [x] Review within the Chief Investigator's institution or host organisation
- [x] Review within the research team
- [ ] Review by educational supervisor
- [ ] Other

Justify and describe the review process and outcome. If the review has been undertaken but not seen by the researcher, give details of the body which has undertaken the review:
As part of the funding process, the funding body have reviewed this project.

Academics at Swansea University and consultants in ABMU HB have also reviewed the project.

For all studies except non-doctoral student research, please enclose a copy of any available scientific critique reports, together with any related correspondence.

For non-doctoral student research, please enclose a copy of the assessment from your educational supervisor/ institution.

A56. How have the statistical aspects of the research been reviewed? *Tick as appropriate:*

- [x] Review by independent statistician commissioned by funder or sponsor
- [ ] Other review by independent statistician
- [ ] Review by company statistician
- [x] Review by a statistician within the Chief Investigator’s institution

- [x] Review by a statistician within the research team or multi−centre group
- [ ] Review by educational supervisor
- [ ] Other review by individual with relevant statistical expertise
- [ ] No review necessary as only frequencies and associations will be assessed – details of statistical input not required

In all cases please give details below of the individual responsible for reviewing the statistical aspects. If advice has been provided in confidence, give details of the department and institution concerned.

	Title	Forename/Initials	Surname
	Dr	S.E.	Jordan

Department	College of Human and Health Sciences
Institution	Swansea University
Work Address	Swansea University
	College of Human and Health Sciences
	Singleton Park
Post Code	Sa2 8PP
Telephone	01792518541
Fax	
Mobile	
E-mail	s.e.jordan@swansea.ac.uk

Please enclose a copy of any available comments or reports from a statistician.

A57. What is the primary outcome measure for the study?

There are two main outcomes for this study. Initially, they are problems found and actions taken at each step in the trial. Each step is seperated by one month and data will be collected using the ADR profile and case note review.

A58. What are the secondary outcome measures? *(if any)*

This project will be used in the application for a feasibility study to assess:
1) Establish rates of recruitment, retention, compliance and cross-over
2) Ensure feasibility of reporting changes in documentation for:
a. Amelioration of problems found using the profile.
b. Problems found and actions taken using the profile.
c. Medication review/ changes.
d. Patients with different severity of illness.
3) Develop clinical endpoints for a full trial, such as measures to capture changes in patients' functioning
4) Calculate intra-cluster correlation coefficient (ICC), and any time delay to patient benefits
5) Explore the basis for cost-effectiveness analysis
6) Report views of care home staff

A59. What is the sample size for the research? *How many participants/samples/data records do you plan to study in total? If there is more than one group, please give further details below.*

Total UK sample size:	87
Total international sample size (including UK):	
Total in European Economic Area:	

Further details:
To allow for potentiall losses, up to 6 care home residents will be recruited, each with up to 12 participants.

Up to 15 interviews will be conducted with staff.

A60. How was the sample size decided upon? *If a formal sample size calculation was used, indicate how this was done, giving sufficient information to justify and reproduce the calculation.*

Using a stepped wedge design allows the number of clusters needed to be lower (Kotz et al. 2012), and 30 participants are considered sufficient in pilot studies (Lancaster et al. 2004). Based on our previous work exploring nurse-led medication monitoring in private care homes we found:
The unit of clustering is the care home, rather than the homes' owner/ employing organisation. There are similarities within each organisation, such as documentation styles; however, each home had its own staff and patient characteristics. When extending the study to local authority run homes, the home, rather than the owners is the only feasible unit of clustering.

A61. Will participants be allocated to groups at random?

○ Yes ◉ No

A62. Please describe the methods of analysis (statistical or other appropriate methods, e.g. for qualitative research) by which the data will be evaluated to meet the study objectives.

Data will be extracted from patient case note records to capture the impact of medication monitoring and behavioural and functional assessments. Data will be grouped by date to establish nursing / medical care at each stepped wedge point.

Marie Gabe, Louise Newson, Sherill Snelgrove and Sue Jordan are responsible for this data collection and will review:
- Number and nature of problems related to adverse effects of antipsychotic, antidepressant and anti-epileptic medication documented as present, actioned or discussed with prescribers
- Follow-up for clinical gain
- drug prescription and administration records, including indications for treatment
- record of functional status, such as Bristol's activities of daily living (ADL) measure, and behavioural or psychological symptoms of dementia
- use of non-pharmacological interventions
- other evidence of clinical change and endpoints

Statistical analysis will be performed using IBM SPSS (SPSS Inc., Chicago, USA). Analysis will be largely descriptive. Time trends will be explored.

ICC, coefficient of variation and sample size for a full trial will be calculated from the variance between and within groups for changes in numbers of problems actioned.

Resource use and clinical gains (if any) will be reported as a basis for cost-effectiveness analysis
Time taken and costs incurred: assessed by completion of pro-formas and discussion with care staff to determine whether a subsequent study will be able to assess cost-effectiveness.

Interviews / group meetings will be explored descriptively to look for dominant trends in the gathered information. During each interview a content validity index will be completed. Interviews will be audio-taped, transcribed and subjected to content analysis.

6. MANAGEMENT OF THE RESEARCH

A63. Other key investigators/collaborators. *Please include all grant co–applicants, protocol co–authors and other key members of the Chief Investigator's team, including non-doctoral student researchers.*

	Title	Forename/Initials	Surname
	Dr	M.E.	Gabe
Post	Research assistant		
Qualifications	PhD (Health Science, 2012); Bachelor of Science (Joint Honours, Biology & Psychology); Bachelor of Nursing (Honours)		

Employer	Swansea University
Work Address	College of Human and Health Sciences<br>Swansea University<br>Singleton Park
Post Code	SA2 8PP
Telephone	01792518568
Fax	
Mobile	07846814571
Work Email	m.gabe@swansea.ac.uk

	Title	Forename/Initials	Surname
	Mr	G	Paynes
Post	Qualified mental health nurse and nurse tutor		
Qualifications	PGCE registered with NMC as LPE, Masters Degree in Nursing, Honours Degree in Nursing 2:1,		
Employer	Swansea University		
Work Address	College of Human and Health Sciences<br>Swansea University<br>Singleton Park		
Post Code	SA2 8PP		
Telephone	01792 423827		
Fax			
Mobile			
Work Email	g.panes@swansea.ac.uk		

	Title	Forename/Initials	Surname
	Professort	I	Russell
Post	Professor of Clinical Trials, Director West Wales Organisation for Rigorous Trials in Health (WWORTH)		
Qualifications	Cambridge (MA in Mathematics)<br>Birmingham (MSc in Statistics)<br>Essex (PhD inHealth Services Research)<br>Aberdeen (Hon DSc in Health Services Research)		
Employer	Swansea University		
Work Address	College of Medicine<br>Swansea University<br>Singleton Park		
Post Code	SA2 8PP		
Telephone	01792 602939		
Fax			
Mobile			
Work Email	i.t.russell@swansea.ac.uk		

	Title	Forename/Initials	Surname
	Professor	P	Huxley
Post	Professor of Social Work and Social Care; & Director of the Centre for Social Work and Social Care Research		
Qualifications	Sociology BA (Hons) 2ii. University of Reading 1968<br>Psychiatric Social Work CQSW. University of Manchester 1971<br>Psychiatric Social Work MSc. University of Manchester 1974<br>PhD Social versus clinical prediction. University of Manchester 1978		
Employer	Swansea University		
Work Address	Centre for Social Work and Social Care Research		

	Swansea University
	Singleton Park
Post Code	SA2 8PP
Telephone	01792602548
Fax	
Mobile	
Work Email	P.J.Huxley@swansea.ac.uk

	Title Forename/Initials Surname<br>Professor M Dennis
Post	Professor of Liaison and Old Age Psychiatry.
Qualifications	MB BCh (Wales, 1983) MD (Leicester, 2003) MRCPsych (1988)<br>GMC Registration No: 2826549, on Specialist Register for Old Age Psychiatry and General Psychiatry
Employer	Swansea University and ABM University Health Board NHS Trust
Work Address	College of Medicine, ILS-2
	Swansea University
	Singleton Park
Post Code	SA2 8PP
Telephone	01792 602145
Fax	
Mobile	
Work Email	m.s.dennis@swansea.ac.uk

	Title Forename/Initials Surname<br>Professor C Phillips
Post	Professor of Health Economics
Qualifications	BSc.(Econ), MSc. (Econ), PhD
Employer	Swansea University
Work Address	College of Human and Health Sciences
	Swansea University
	Singleton Park
Post Code	SA2 8PP
Telephone	01792295729
Fax	
Mobile	
Work Email	C.J.Phillips@swansea.ac.uk

	Title Forename/Initials Surname<br>Dr S Snelgrove
Post	Lecturer in Nursing and Psychology applied to Health Care, College of Human and Health Sciences, Swansea University
Qualifications	PhD, M.Phil, BSc (Hons), RGN, PGCE
Employer	Swansea University
Work Address	College of Human and Health Sciences
	Swansea University
	Singleton Park
Post Code	SA2 8PP
Telephone	01792 513466
Fax	

Mobile	
Work Email	S.R.Snelgrove@swansea.ac.uk

	Title	Forename/Initials	Surname
	Dr	R	Colgate

Post	Consultant in Old Age Liaison Psychiatry
Qualifications	MB BCh. GMC Registration 3168518
Employer	ABMU HB
Work Address	Princess of Wales Hospital<br>Coity Road<br>Bridgend
Post Code	CF31 1RQ
Telephone	01656 752752 x2252
Fax	01656 754122
Mobile	
Work Email	Robert.Colgate@wales.nhs.uk

	Title	Forename/Initials	Surname
	Dr	C	Baker

Post	Researcher
Qualifications	PhD, University of Wales, Swansea, Bachelor of Economic and Social Studies Bsc(Econ) Hons (University of Wales, Swansea), Further Education Teaching Certificate. (FETC). WJEC 1997, General Nurse 1974.
Employer	Swansea University
Work Address	College of Human and Health Sciences and College of Medicine<br>Swansea University<br>Singleton Park
Post Code	SA2 8PP
Telephone	01792 602632
Fax	01792 295856
Mobile	
Work Email	c.m.baker@swansea.ac.uk

	Title	Forename/Initials	Surname
	Ms	L	Newson

Post	Pre-registration nuring student (mental health branch)
Qualifications	BSc (Psychology Swansea University)
Employer	Swansea University
Work Address	College of Human and Health Sciences<br>Singleton Park<br>Swansea University
Post Code	SA2 8PP
Telephone	07969247294
Fax	
Mobile	
Work Email	516171@swansea.ac.uk

A64. Details of research sponsor(s)

A64-1. Sponsor

Lead Sponsor

Status:
- ◯ NHS or HSC care organisation
- ◉ Academic
- ◯ Pharmaceutical industry
- ◯ Medical device industry
- ◯ Local Authority
- ◯ Other social care provider (including voluntary sector or private organisation)
- ◯ Other

If Other, please specify:

Commercial status: Non-Commercial

Contact person

Name of organisation	Swansea University
Given name	Ceri
Family name	Jones
Address	Swansea University
Town/city	Swansea
Post code	SA2 8PP
Country	UNITED KINGDOM
Telephone	01792295412
Fax	
E-mail	c.d.jones@swansea.ac.uk

Is the sponsor based outside the UK?

◯ Yes ◉ No

Under the Research Governance Framework for Health and Social Care, a sponsor outside the UK must appoint a legal representative established in the UK. Please consult the guidance notes.

A65. Has external funding for the research been secured?

- [x] Funding secured from one or more funders
- [ ] External funding application to one or more funders in progress
- [ ] No application for external funding will be made

What type of research project is this?

- ◉ Standalone project
- ◯ Project that is part of a programme grant
- ◯ Project that is part of a Centre grant
- ◯ Project that is part of a fellowship/ personal award/ research training award
- ◯ Other

Other – please state:

Please give details of funding applications.

Organisation	Wales School for Primary Care Research
Address	Department of Primary Care and Public Health<br>Cardiff University<br>School of Medicine
Post Code	CF14 4XN
Telephone	++ 44 29 2068 7168
Fax	
Mobile	
Email	wspcr@cardiff.ac.uk

Funding Application Status: (●) Secured ( ) In progress

Amount: £20670.74

Duration
Years: 1
Months: 0

If applicable, please specify the programme/ funding stream:

What is the funding stream/ programme for this research project?

A67. Has this or a similar application been previously rejected by a Research Ethics Committee in the UK or another country?

( ) Yes (●) No

Please provide a copy of the unfavourable opinion letter(s). You should explain in your answer to question A6-2 how the reasons for the unfavourable opinion have been addressed in this application.

A68. Give details of the lead NHS R&D contact for this research:

	Title Forename/Initials Surname
Organisation	NISCHR Permissions Co-ordinating Unit
Address	Powys teaching Health Board<br>Room 12, Monnow Ward,<br>Bronllys Hospital, Brecon
Post Code	LD3 0LS
Work Email	NISCHR.PCU.Allwales@wales.nhs.uk
Telephone	01874 712450
Fax	
Mobile	

Details can be obtained from the NHS R&D Forum website: http://www.rdforum.nhs.uk

A69-1. How long do you expect the study to last in the UK?

Planned start date: 01/02/2013
Planned end date: 31/01/2014
Total duration:
Years: Months: 11 Days: 30

A71-2. Where will the research take place? *(Tick as appropriate)*

- [ ] England
- [ ] Scotland
- [x] Wales
- [ ] Northern Ireland
- [ ] Other countries in European Economic Area

Total UK sites in study

Does this trial involve countries outside the EU?
( ) Yes (•) No

A72. What host organisations (NHS or other) in the UK will be responsible for the research sites? *Please indicate the type of organisation by ticking the box and give approximate numbers of planned research sites:*

[ ] NHS organisations in England	
[x] NHS organisations in Wales	5
[ ] NHS organisations in Scotland	
[ ] HSC organisations in Northern Ireland	
[ ] GP practices in England	
[ ] GP practices in Wales	
[ ] GP practices in Scotland	
[ ] GP practices in Northern Ireland	
[ ] Social care organisations	
[ ] Phase 1 trial units	
[ ] Prison establishments	
[ ] Probation areas	
[x] Independent hospitals	1
[ ] Educational establishments	
[ ] Independent research units	
[ ] Other (give details)	
Total UK sites in study:	6

A75-1. What arrangements will be made to review interim safety and efficacy data from the trial? Will a formal data monitoring committee or equivalent body be convened?

Ongoing safety & efficacy will be examined by Marie Gabe & Sue Jordan, and discussed with the care home and consultants (Professor Dennis & Dr. Colgate). Any concerns will be dealt with immediately, if necessary the study will be paused / suspended.

If a formal DMC is to be convened, please forward details of the membership and standard operating procedures to the Research Ethics Committee when available. The REC should also be notified of DMC recommendations and receive summary reports of interim analyses.

A75-2. What are the criteria for electively stopping the trial or other research prematurely?

Any member of the research team or care home staff can raise concerns to discontinue the study. The research team will consider any reports and work with the NHS research ethics committee.

A76. Insurance/ indemnity to meet potential legal liabilities

***Note:* in this question to NHS indemnity schemes include equivalent schemes provided by Health and Social Care (HSC) in Northern Ireland**

A76-1. What arrangements will be made for insurance and/or indemnity to meet the potential legal liability of the sponsor(s) for harm to participants arising from the management of the research? *Please tick box(es) as applicable.*

Note: Where a NHS organisation has agreed to act as sponsor or co-sponsor, indemnity is provided through NHS schemes. Indicate if this applies (there is no need to provide documentary evidence). For all other sponsors, please describe the arrangements and provide evidence.

☐ NHS indemnity scheme will apply (NHS sponsors only)

☑ Other insurance or indemnity arrangements will apply (give details below)

Swansea University, as the sponsor of the project will provide insurance and indemnity cover. Swansea University, Singleton Campus. SA2 8PP.

Please enclose a copy of relevant documents.

A76-2. What arrangements will be made for insurance and/ or indemnity to meet the potential legal liability of the sponsor(s) or employer(s) for harm to participants arising from the design of the research? *Please tick box(es) as applicable.*

Note: Where researchers with substantive NHS employment contracts have designed the research, indemnity is provided through NHS schemes. Indicate if this applies (there is no need to provide documentary evidence). For other protocol authors (e.g. company employees, university members), please describe the arrangements and provide evidence.

☐ NHS indemnity scheme will apply (protocol authors with NHS contracts only)

☑ Other insurance or indemnity arrangements will apply (give details below)

Swansea University, as the sponsor of the project will provide insurance and indemnity cover. Swansea University, Singleton Campus. SA2 8PP.

Please enclose a copy of relevant documents.

A76-3. What arrangements will be made for insurance and/ or indemnity to meet the potential legal liability of investigators/collaborators arising from harm to participants in the conduct of the research?

Note: Where the participants are NHS patients, indemnity is provided through the NHS schemes or through professional indemnity. Indicate if this applies to the whole study (there is no need to provide documentary evidence). Where non-NHS sites are to be included in the research, including private practices, please describe the arrangements which will be made at these sites and provide evidence.

☐ NHS indemnity scheme or professional indemnity will apply (participants recruited at NHS sites only)

☑ Research includes non-NHS sites (give details of insurance/ indemnity arrangements for these sites below)

Swansea University, as the sponsor of the project will provide insurance and indemnity cover. Swansea University, Singleton Campus. SA2 8PP.

Please enclose a copy of relevant documents.

A77. Has the sponsor(s) made arrangements for payment of compensation in the event of harm to the research

participants where no legal liability arises?

(•) Yes ( ) No

If Yes, please give details of the compensation policy:
1. EMPLOYERS' LIABILITY
Certificate No.
Y016458QBE0112A/028
Period of Cover
1 August 2012 to 31 July 2013
Limit of Indemnity
£50,000,000 any one event unlimited in the aggregate.
Includes
Indemnity to Principals
Cover provided by
QBE Insurance (Europe) Limited and Excess Insurers.
2. PUBLIC AND PRODUCTS LIABILITY
Certificate of Entry No.
UM028/95
Period of Cover
1 August 2012 to 31 July 2013
Includes
Indemnity to Principals
Limit Of Indemnity
£50,000,000 any one event and in the aggregate in respect of Products Liability and unlimited in the aggregate in respect of Public Liability.
Cover provided by
U.M. Association Limited and Excess Cover Providers led by QBE Insurance (Europe) Limited

Please enclose a copy of relevant documents.

B. All research other than CTIMPs

In this sub-section, an adult means a person aged 16 or over.

B1. What impairing condition(s) will the participants have?

The study must be connected to this condition or its treatment.

Patients with dementia and dementia sub-types

B2. Justify the inclusion of adults unable to consent for themselves. It should be clear why the research could not be carried out as effectively if confined to adults capable of giving consent.

The study will not presume that all participants lack capacity to consent, however, patients with dementia may need to be carefully considered in terms of their ability to give informed consent.

Potential participants are being asked to consent to the researcher accessing their case notes to look for information on their medication experience. The nurse will use the ADR profile and related assessments as part of their usual care. Working with patients with dementia poses a potential dilemma in obtaining consent. The nurses will be asked to select relevant patients based on the inclusion / exclusion criteria. Where necessary legal guardians may be asked for permission. The qualified staff will therefore be mindful that the participants' capacity to consent is carefully considered when consent is sought, to safeguard their interest and autonomy. Any doubts of the patients / guardians ability to give informed consent will result in postponing or terminating involvement in the project.

The work is directed towards people with dementia, in part, in response to needs highlighted in the literature (Banerjee 2009).

B3. Who in the research team will decide whether or not the participants have the capacity to give consent? What training/experience will they have to enable them to reach this decision?

The qualified nursing staff who meet the following criteria:
Registered care home staff
Qualified nursing staff
Familiar with the Mental Capacity Act (2005) or attended GCP training

B4. Does the research have the potential to benefit participants who are unable to consent for themselves?

(●) Yes ( ) No

If Yes, please indicate the nature of this benefit. You may refer back to your answer to Question A24.

Participants will be routinely asked a comprehensive list of questions that will ensure that all pertinent information has been gained on their physical well-being. Previous evidence (Jordan 2002, Jordan et al 2002) suggests that patients' quality of life may be improved and potentially life-threatening problems may be identified and addressed expeditiously, when this tool is operationalised.

B5. Will the research contribute to knowledge of the causes or the treatment or care of persons with the same impairing condition (or a similar condition)?

(●) Yes ( ) No

If Yes, please explain how the research will achieve this:

Routine administration of structured, standardised questionnaires, checklists or diaries is a low risk strategy to recognise and action the problems caused by adverse effects of medication (Tierney 2003), possibly in conjunction with care pathways. Our previous work indicates that introduction of structured data collection by nurses facilitates the recognition and treatment of previously
undisclosed and unsuspected problems.

B6. Will the research involve any foreseeable risk or burden for these participants, or interfere in any way with their

freedom of action or privacy?

(•) Yes ( ) No

If Yes, please give an assessment below. Highlight any risk, burden or discomfort specific to these participants and say what will be done to minimise it. You may refer back to your answers to Questions A22 and A23.

Our study is client-centred and all those involved, whether they choose to withdraw or not, will receive the utmost care. Few risks are perceived to participants as they will continue to receive the usual and standard care of the care home. However, participants may have extra contact with the nursing staff when completing the West Wales ADR Profile (up to five completed profiles per participant) and related assessments (6 completed assessments for each participant). Therefore, all nurse-patient consultations will take place at a time and pace to suit the participant.

If the participant becomes anxious as a result of the administration of the West Wales ADR Profile, the nursing staff will be able to discontinue / pause completion of the profile. When the participant feels able, the nurse and participant will be able to revisit the profile. The trained staff in the care home are prepared to counsel and support participants throughout the project.

Participants will be supported to discontinue involvement in the project should they express the wish to do so. Usual care will remain upon completion of the project.

Questions B7 and B8 apply to any participants recruited in England and Wales.

B7. What arrangements will be made to identify and consult persons able to advise on the presumed wishes and feelings of participants unable to consent for themselves and on their inclusion in the research?

The guardians of potential participants will be approached for any individual for whom capacity to consent is lacking (as determined by the qualified staff). The guardian will usually be known to care home staff or will be identified from the patients' case notes. As with all participants, the guardian will be given written and oral information, and can take up to two weeks to decided if he/she believes that the study is appropriate for the participant. Guardians will know the patient well but will not be acting in a professional or paid capacity.

Please enclose a copy of the written information to be provided to consultees. This should describe their role under section 32 of the Mental Capacity Act and provide information about the research similar to that which might be given to participants able to consent for themselves.

B8. Is it possible that a participant requiring urgent treatment might need to be recruited into research before it is possible to identify and consult a person under B7?

( ) Yes (•) No

If Yes, say whether arrangements will be made instead to seek agreement from a registered medical practitioner and outline these arrangements. Or, if this is also not feasible, outline how decisions will be made on the inclusion of participants and what arrangements will be made to seek consent from the participant (if capacity has been recovered) or advice from a consultee as soon as practicable thereafter.

B9. What arrangements will be made to continue to consult such persons during the course of the research where necessary?

Guardians will only be consulted if the participant experiences an adverse event. Guardian information will be accessed from the participants' notes. Guardian personal information will not be kept. Guardians will be notified that executive summaries will be available in the care home at the end of the study.

B10. What steps will you take, if appropriate, to provide participants who are unable to consent for themselves with information about the research, and to consider their wishes and feelings?

Participants who are considered to lack the capacity to consent will continue to receive the usual care of the care home. In addition, care home staff will also speak to them about any problems with their medication, as per the ADR profile.

B11. Is it possible that the capacity of participants could fluctuate during the research? How would this be handled?

This is not likely, the research will take one year to complete, but involvement at care homes is only likely to be for 5

months. Throughout the study, the qualified staff, responsible for obtaining consent, will continue to monitor participants for any changes in their ability to consent.

B12-1. What will be the criteria for withdrawal of participants?

Guardians can choose to withdraw their consent at any time without reason. Similarly, participants can also withdraw, without reason, at any time. All will be reassured that this will not affect the care received by the participant.

B13. Describe what steps will be taken to ensure that nothing is done to which participants appear to object (unless it is to protect them from harm or minimise pain or discomfort).

The healthcare staff will be mindful of the participant's right to decline care. Staff will ensure that participants are fully informed of the care they are receiving and the reasons for it.

Participants will not be involved during the case note review.

B14. Describe what steps will be taken to ensure that nothing is done which is contrary to any advance decision or statement by the participant?

The participants' right to self-determination is important and will outweigh the needs of the project. Guardians will similarly be asked to consider any advance decisions made by participants.

PART C: Overview of research sites

Please enter details of the host organisations (Local Authority, NHS or other) in the UK that will be responsible for the research sites. *For NHS sites, the host organisation is the Trust or Health Board. Where the research site is a primary care site, e.g. GP practice, please insert the host organisation (PCT or Health Board) in the Institution row and insert the research site (e.g. GP practice) in the Department row.*

Research site		Investigator/ Collaborator/ Contact	
Institution name	Fieldbay	Title	Dr
Department name	Heathfield Lodge	First name/ Initials	S.E
Street address	35 Heathfield	Surname	Jordan
Town/city	Swansea		
Post Code	SA1 6HD		
Institution name	Monsktone House	Title	Dr
Department name	Residential Care	First name/ Initials	S.E.
Street address	1 Locks Common Road	Surname	Jordan
Town/city	Porthcawl		
Post Code	CF36 3HU		
Institution name	Danygraig	Title	Dr
Department name	Care Home	First name/ Initials	S.E.
Street address	Bridgend Road	Surname	Jordan
Town/city	Porthcawl		
Post Code	CF36 5SR		

PART D: Declarations

D1. Declaration by Chief Investigator

1. The information in this form is accurate to the best of my knowledge and belief and I take full responsibility for it.

2. I undertake to abide by the ethical principles underlying the Declaration of Helsinki and good practice guidelines on the proper conduct of research.

3. If the research is approved I undertake to adhere to the study protocol, the terms of the full application as approved and any conditions set out by review bodies in giving approval.

4. I undertake to notify review bodies of substantial amendments to the protocol or the terms of the approved application, and to seek a favourable opinion from the main REC before implementing the amendment.

5. I undertake to submit annual progress reports setting out the progress of the research, as required by review bodies.

6. I am aware of my responsibility to be up to date and comply with the requirements of the law and relevant guidelines relating to security and confidentiality of patient or other personal data, including the need to register when necessary with the appropriate Data Protection Officer. I understand that I am not permitted to disclose identifiable data to third parties unless the disclosure has the consent of the data subject or, in the case of patient data in England and Wales, the disclosure is covered by the terms of an approval under Section 251 of the NHS Act 2006.

7. I understand that research records/data may be subject to inspection by review bodies for audit purposes if required.

8. I understand that any personal data in this application will be held by review bodies and their operational managers and that this will be managed according to the principles established in the Data Protection Act 1998.

9. I understand that the information contained in this application, any supporting documentation and all correspondence with review bodies or their operational managers relating to the application:

   - Will be held by the REC (where applicable) until at least 3 years after the end of the study; and by NHS R&D offices (where the research requires NHS management permission) in accordance with the NHS Code of Practice on Records Management.
   - May be disclosed to the operational managers of review bodies, or the appointing authority for the REC (where applicable), in order to check that the application has been processed correctly or to investigate any complaint.
   - May be seen by auditors appointed to undertake accreditation of RECs (where applicable).
   - Will be subject to the provisions of the Freedom of Information Acts and may be disclosed in response to requests made under the Acts except where statutory exemptions apply.

10. I understand that information relating to this research, including the contact details on this application, may be held on national research information systems, and that this will be managed according to the principles established in the Data Protection Act 1998.

11. Where the research is reviewed by a REC within the UK Health Departments Research Ethics Service, I understand that the summary of this study will be published on the website of the National Research Ethics Service (NRES), together with the contact point for enquiries named below. Publication will take place no earlier than 3 months after issue of the ethics committee’s final opinion or the withdrawal of the application.

Contact point for publication*(Not applicable for R&D Forms)*

NRES would like to include a contact point with the published summary of the study for those wishing to seek further information. We would be grateful if you would indicate one of the contact points below.

(●) Chief Investigator

( ) Sponsor

- ◯ Study co-ordinator
- ◯ Student
- ◯ Other – please give details
- ◯ None

Access to application for training purposes *(Not applicable for R&D Forms)*

Optional – please tick as appropriate:

☐ I would be content for members of other RECs to have access to the information in the application in confidence for training purposes. All personal identifiers and references to sponsors, funders and research units would be removed.

Signature: ....

Print Name: Sue Jordan

Date: *(dd/mm/yyyy)*

D2. Declaration by the sponsor's representative

If there is more than one sponsor, this declaration should be signed on behalf of the co–sponsors by a representative of the lead sponsor named at A64-1.

I confirm that:

1. This research proposal has been discussed with the Chief Investigator and agreement in principle to sponsor the research is in place.

2. An appropriate process of scientific critique has demonstrated that this research proposal is worthwhile and of high scientific quality.

3. Any necessary indemnity or insurance arrangements, as described in question A76, will be in place before this research starts. Insurance or indemnity policies will be renewed for the duration of the study where necessary.

4. Arrangements will be in place before the study starts for the research team to access resources and support to deliver the research as proposed.

5. Arrangements to allocate responsibilities for the management, monitoring and reporting of the research will be in place before the research starts.

6. The duties of sponsors set out in the Research Governance Framework for Health and Social Care will be undertaken in relation to this research.

7. Where the research is reviewed by a REC within the UK Health Departments Research Ethics Service, I understand that the summary of this study will be published on the website of the National Research Ethics Service (NRES), together with the contact point for enquiries named in this application. Publication will take place no earlier than 3 months after issue of the ethics committee's final opinion or the withdrawal of the application.

Signature: ....

Print Name:

Post:

Organisation:

Date: *(dd/mm/yyyy)*
