# Supplementary material for: Nurse-Led Medicines' Monitoring for Patients with Dementia in Care Homes: A Pragmatic Cohort Stepped Wedge Cluster Randomised Trial
Source: PLoS One. 2015 Oct 13;10(10):e0140203. doi: 10.1371/journal.pone.0140203 (PMC4603896; doi:10.1371/journal.pone.0140203)
Supplement: S1 Table — This is the S1 table legend reporting descriptive data in full. (DOCX) [file pone.0140203.s005.docx]

**S1 Table. Total number of problems found per participant at each step and in each site.**

| Site | Step 1. Total number of problems found | Step 2.  Total number of problems found | Step 3. Total number of problems found | Step 4. Total number of problems found | Step 5. Total number of problems found | Step 6. Total number of problems found |
| --- | --- | --- | --- | --- | --- | --- |
| **5:** n  Mean [SD]  Median  25^th^ - 75^th^ centile  Full range | 8  6.38 [2.00]  6.00  5.00-6.75  5-11 | 9  6.78 [1.92]  6.00  6.00-7.50  4-11 | 10  7.50 [2.32]  7.00  5.75-8.75  5-12 | 9  7.70 [3.65]  7.50  5.75-9.75  1-14 | 9  7.40 [3.89]  8.00  4.75-8.25  1-16 | **9**  **16.50 [6.59]**  **16.50**  **14.00-22.25**  **1-24** |
| **4:** n  Mean [SD]  Median  25^th^ - 75^th^ centile  Full range | 10  9.10 [3.81]  9.50  6.50-12.50  2-14 | 10  6.20 [2.49]  6.50  4.00-8.25  2-10 | 10  7.00 [2.63]  7.50  5.50-9.00  2-11 | 9  4.56 [2.30]  4.00  2.50-7.00  2-8 | **9**  **18.33 [7.57]**  **21.00**  **10.50-25.50**  **7-27** | **9**  **19.11[6.79]**  **22.00**  **13.00-25.50**  **0-5** |
| **3:** n  Mean [SD]  Median  25^th^ - 75^th^ centile  Full range | 5  8.20 [4.09]  6.00  5.50-12.00  5-15 | 5  6.60 [2.30]  7.00  4.50-8.50  3-9 | 5  6.80 [4.32]  6.00  3.50-10.50  1-13 | **5**  **10.60 [2.07]**  **11.00**  **8.50-12.50**  **8-13** | **5**  **9.40 [1.14]**  **9.00**  **8.50-10.50**  **8-11** | **5**  **12.20[ 2.39]**  **12.00**  **10.00-14.50**  **9-15** |
| **2:** n  Mean [SD]  Median  25^th^ - 75^th^ centile  Full range | 8  8.13 [3.72]  8.00  4.50-11.75  3-13 | 8  5.38 [3.58]  5.50  1.50-8.75  1-10 | **8**  **15.75 [6.94]**  **16.00**  **10.75-20.00**  **5-28** | **8**  **12.75 [5.90]**  **13.50**  **7.25-14.75**  **5-24** | **8**  **12.88 [6.31]**  **13.00**  **6.50-19.00**  **5-22** | **8**  **14.38 [5.73]**  **13.00**  **12-14**  **9-28** |
| **1:** n  Mean [SD]  Median  25^th^ - 75^th^ centile  Full range | 10  10.30 [2.21]  10.50  8.00-11.50  7-14 | **10**  **17.30 [6.08]**  **16.50**  **11.50-24.25**  **10-25** | **10**  **17.30 [6.09]**  **18.50**  **12.50-22.25**  **6-25** | **10**  **17.00 [5.25]**  **17.50**  **14.00-20.25**  **6-26** | **10**  **16.50 [5.19]**  **17.00**  **14.50-20.00**  **5-23** | **10**  **17.10[ 4.61]**  **18.00**  **15.75-19.50**  **6-23** |

Bold text indicates roll-out of medicines’ monitoring and Profile administration 1 month before these data collection points. N is the number of service users in the site.

One participant from site 4 passed away between steps 3 & 4. One participant from site 5 was hospitalised between steps 3 & 4. Participants joined site 5 at steps 2 and 3. Problems explored are listed in Table 5 and on the Profile, appendix S1.
