# Supplementary material for: Nurse-Led Medicines' Monitoring for Patients with Dementia in Care Homes: A Pragmatic Cohort Stepped Wedge Cluster Randomised Trial
Source: PLoS One. 2015 Oct 13;10(10):e0140203. doi: 10.1371/journal.pone.0140203 (PMC4603896; doi:10.1371/journal.pone.0140203)
Supplement: S2 Table — This is the S2 table legend reporting descriptive data in full. (DOCX) [file pone.0140203.s006.docx]

**S2 Table. Total number of actions taken per participant at each step in each site**

| Site | Step 1. Total number of actions taken | Step 2.  Total number of actions taken | Step 3. Total number of actions taken | Step 4. Total number of actions taken | Step 5. Total number of actions taken | Step 6. Total number of actions taken |
| --- | --- | --- | --- | --- | --- | --- |
| **5:** n  Mean [SD]  Median  25^th^ - 75^th^ centile  Full range | 8  4.25 [1.67]  4.00  3.00-4.75  3-8 | 9  4.89 [1.90]  4.00  3.50-6.00  3-9 | 10  5.30 [2.41]  4.50  3.75-6.75  3-10 | 9  5.00 [2.83]  5.00  3.75-5.25  1-12 | 9  4.40 [2.22]  4.50  3.25-5.50  1-8 | **9**  **10.70[4.97]**  **11.00**  **9.25-12.25**  **91-21** |
| **4:** n  Mean [SD]  Median  25^th^ - 75^th^ centile  Full range | 10  8.50 [3.44]  8.50  6.50-12.00  2-13 | 10  5.90 [2.47]  5.50  4.00-8.25  2-10 | 10  6.60 [2.27]  7.00  5.50-8.25  2-9 | 9  4.44 [2.24]  4.00  2.50-6.50  2-8 | **9**  **12.00 [4.61]**  **12.00**  **7.00-16.00**  **6-19** | **9**  **12.78 [4.68]**  **14.00**  **7.50-16.50**  **6-19** |
| **3:** n  Mean [SD]  Median  25^th^ - 75^th^ centile  Full range | 5  7.00 [2.92]  6.00  4.50-10.00  4-11 | 5  5.80 [1.92]  6.00  4.00-7.50  3-8 | 5  6.00 [4.18]  5.00  2.50-10.00  1-12 | **5**  **4.60 [1.34]**  **4.00**  **3.50-6.00**  **3-6** | **5**  **4.60 [1.52]**  **4.00**  **3.50-6.00**  **3-7** | **5**  **6.20 [2.17]**  **5.00**  **4.50-8.50**  **0-6** |
| **2:** n  Mean [SD]  Median  25^th^ - 75^th^ centile  Full range | 8  7.25 [3.37]  7.50  4.25-10.75  2-11 | 8  5.00 [3.12]  5.50  1.50-8.00  1-8 | **8**  **11.50 [4.38]**  **12.00**  **9.50-14.25**  **3-18** | **8**  **8.88 [4.61]**  **10.00**  **4.75-10.75**  **2-17** | **8**  **7.88 [5.17]**  **6.50**  **3.25-13.00**  **3-16** | **8**  **10.13 [5.19]**  **9.00**  **8.25-10.50**  **4-22** |
| **1:** n  Mean [SD]  Median  25^th^ - 75^th^ centile  Full range | 10  9.30 [2.63]  9.50  7.00-11.50  5-13 | **10**  **8.60 [2.99]**  **8.00**  **6.00-10.75**  **5-14** | **10**  **9.80 [3.62]**  **10.50**  **7.25-13.00**  **3-14** | **10**  **10.10 [3.41]**  **10.50**  **8.25-12.25**  **4-16** | **10**  **10.80 [4.57]**  **10.00**  **8.25-14.50**  **3-18** | **10**  **11.70[4.72]**  **11.50**  **8.50-15.25**  **4-20** |

Bold text indicates roll-out of medicines’ monitoring and Profile use. N = number of service users in the site.

Problems explored are listed in Table 5 and on the Profile, appendix S1
