# Supplementary material for: Nurse-Led Medicines' Monitoring for Patients with Dementia in Care Homes: A Pragmatic Cohort Stepped Wedge Cluster Randomised Trial
Source: PLoS One. 2015 Oct 13;10(10):e0140203. doi: 10.1371/journal.pone.0140203 (PMC4603896; doi:10.1371/journal.pone.0140203)
Supplement: S3 Table — This is the S3 table legend reporting descriptive data in full. (DOCX) [file pone.0140203.s007.docx]

**S3 Table. Number of prescribed medicines in each step for each site**

| Site | Step 1. Number of prescribed medicines | Step 2. Number of prescribed medicines | Step 3. Number of prescribed medicines | Step 4. Number of prescribed medicines | Step 5. Number of prescribed medicines | Step 6. Number of prescribed medicines |
| --- | --- | --- | --- | --- | --- | --- |
| **5:** Sum (n)  Mean [SD]  Median  25^th^ - 75^th^ centile  Full range | 85 (8)  10.63[3.07]  10.50  7.50-13.50  7-15 | 96 (9)  10.67 [3.16]  11.00  7.50-13.00  7-16 | 109 (10)  10.90 [3.28]  11.00  7.75-14.00  6-16 | 105 (9)  11.67 [2.92]  12.00  10.00-14.50  6-15 | 108 (9)  12.00 [3.12]  13.00  9.50-15.00  7-16 | **115 (9)**  **12.78[40.6]**  **13.00**  **9.50-16.00**  **6-19** |
| **4:** Sum (n)  Mean [SD]  Median  25^th^ - 75^th^ centile  Full range | 91 (10)  9.10 [4.23]  8.00  6.50-11.75  4-18 | 93 (10)  9.30 [4.24]  8.00  6.50-12.50  4-18 | 18 (10)  9.60 [3.95]  8.00  7.50-12.50  5-18 | 92 (9)  10.22 [4.30]  8.00  8.00-13.50  5-19 | **98 (9)**  **10.89 [4.83]**  **10.00**  **7.00-14.00**  **6-21** | **101 (9)**  **11.22 [4.06]**  **11.00**  **7.50-15.00**  **7-18** |
| **3:** Sum (n)  Mean [SD]  Median  25^th^ - 75^th^ centile  Full range | 48 (5)  9.60 [1.14]  10.00  8.50-10.50  8-11 | 50 (5)  10.00 [2.00]  10.00  8.00-12.00  8-12 | 49 (5)  9.80 [1.79]  10.00  8.00-11.50  8-12 | **50 (5)**  **10.00 [1.23]**  **10.00**  **9.00-11.00**  **8-11** | **49 (5)**  **9.80 [1.30]**  **10.00**  **8.50-11.00**  **8-11** | **49 (5)**  **9.80 [1.30]**  **10.00**  **8.50-11.00**  **8-11** |
| **2:** Sum (n)  Mean [SD]  Median  25^th^ - 75^th^ centile  Full range | 89 (8)  11.13 [3.91]  10.50  9.00-13.25  6-19 | 89 (8)  11.13 [4.29]  10.50  8.25-13.25  6-20 | **89 (8)**  **11.13 [3.56]**  **10.50**  **9.25-13.25**  **6-18** | **88 (8)**  **11.00 [3.21]**  **10.50**  **9.25-12.75**  **6-17** | **85 (8)**  **10.63 [3.29]**  **10.00**  **8.50-12.50**  **6-17** | **89 (8)**  **11.13 [3.56]**  **9.25-13.00**  **6-18** |
| **1:** Sum (n)  Mean [SD]  Median  25^th^ - 75^th^ centile  Full range | 68 (10)  6.80 [3.36]  6.00  4.00-9.25  2-13 | **72 (10)**  **7.20 [3.33]**  **6.50**  **4.75-9.50**  **2-13** | **72 (10)**  **7.20 [3.49]**  **6.00**  **4.75-9.75**  **2-13** | **71 (10)**  **7.10 [3.35]**  **6.00**  **4.75-9.50**  **2-13** | **70 (10)**  **7.00 [3.50]**  **6.00**  **4.75-9.25**  **2-14** | **84 (10)**  **8.40 [3.98]**  **7.50**  **5.75-12.25**  **2-14** |

Bold text indicates roll-out of medicines’ monitoring.
